# Synthesis, Crystallographic Structure, Theoretical Analysis, Molecular Docking Studies, and Biological Activity Evaluation of Binuclear Ru(II)-1-Naphthylhydrazine Complex

**DOI:** 10.3390/ijms24010689

**Published:** 2022-12-30

**Authors:** Thomas Eichhorn, Franz Kolbe, Stefan Mišić, Dušan Dimić, Ibrahim Morgan, Mohamad Saoud, Dejan Milenković, Zoran Marković, Tobias Rüffer, Jasmina Dimitrić Marković, Goran N. Kaluđerović

**Affiliations:** 1 Department of Engineering and Natural Sciences, University of Applied Sciences Merseburg, Eberhard-Leibnitz-Straße 2, 06217 Merseburg, Germany; 2 Faculty of Physical Chemistry, University of Belgrade, Studentski trg 12-16, 11000 Belgrade, Serbia; 3 Department of Bioorganic Chemistry, Leibniz Institute of Plant Biochemistry, Weinberg 3, 06120 Halle (Saale), Germany; 4 Department of Science, Institute for Information Technologies, University of Kragujevac, Jovana Cvijića bb, 34000 Kragujevac, Serbia; 5 Institute of Chemistry, Chemnitz University of Technology, Straße der Nationen 62, D-09111 Chemnitz, Germany

**Keywords:** Ru(II) complexes, DFT, radical scavenging activity, BSA, hydrazine

## Abstract

Ruthenium(II)–arene complexes have gained significant research interest due to their possible application in cancer therapy. In this contribution two new complexes are described, namely [{RuCl(η^6^-*p*-cymene)}_2_(μ-Cl)(μ-1-*N*,*N′*-naphthyl)]X (X = Cl, **1**; PF_6_, **2**), which were fully characterized by IR, NMR, and elemental microanalysis. Furthermore, the structure of **2** in the solid state was determined by a single crystal X-ray crystallographic study, confirming the composition of the crystals as **2·2MeOH**. The Hirshfeld surface analysis was employed for the investigation of interactions that govern the crystal structure of **2·2MeOH**. The structural data for **2** out of **2·2MeOH** was used for the theoretical analysis of the cationic part [{RuCl(η^6^-*p*-cymene)}_2_(μ-Cl)(μ-1-*N*,*N′*-naphthyl)]^+^ (**2a**) which is common to both **1** and **2**. The density functional theory, at B3LYP/6-31+G(d,p) basis set for H, C, N, and Cl atoms and LanL2DZ for Ru ions, was used for the optimization of the **2a** structure. The natural bond orbital and quantum theory of atoms in molecules analyses were employed to quantify the intramolecular interactions. The reproduction of experimental IR and NMR spectra proved the applicability of the chosen level of theory. The binding of **1** to bovine serum albumin was examined by spectrofluorimetry and molecular docking, with complementary results obtained. Compound **1** acted as a radical scavenger towards DPPH^•^ and HO^•^ radicals, along with high activity towards cancer prostate and colon cell lines.

## 1. Introduction

Cancer is one of the most prominent causes of death in the modern world. As with most human diseases, it is a very complex, multifactorial, and oxidative stress-related disease. The discovery of the anticancer effect of cisplatin in the 60s of the twentieth century was revolutionary in this field and stimulated further investigation of platinum complexes. Despite some clinical success of cisplatin complexes, these compounds inflict some deleterious side effects, such as myelosuppression, peripheral neuropathy, emesis, nephrotoxicity, fatigue, alopecia, or ototoxicity. They also cause inherent resistance to the treatment of some cancer types. Because of this, it is of high importance to create new types of biologically active compounds, which may be suitable for the treatment of cancer [1,2].

Since most of the anticancer compounds under development are ineffective in the treatment of malignant cancers, much attention has been focused on finding more effective and less toxic complexes than the existing pharmaceuticals. Attention has been focused on other Pt group elements, such as palladium, ruthenium, iridium, rhodium, and osmium, and the novel design strategy of metal complexes [3,4,5].

**Figure 1:**
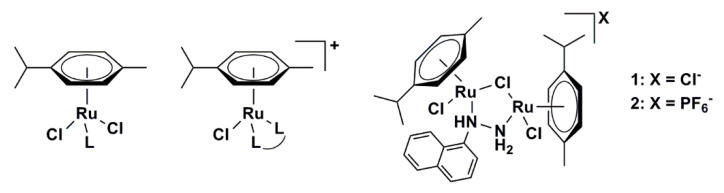
General structure of “half sandwich” Ru(II)-arene compounds and structures of the obtained complexes 1 and 2 (**right**).

Ruthenium complexes are a generation of drugs that could replace the already existing platinum complexes due to the human organism’s higher tolerance even at relatively high ruthenium concentrations as well as better selectivity towards cancerous cells. The discovery of the inhibitory effect of the *fac*-[RuCl_3_(NH_3_)_3_] complex on *Escherichia coli* cell division aroused interest in the use of ruthenium complexes as potential anticancer agents [6,7]. Ruthenium is a transition metal present in +2, +3, and +4 oxidation states with the first two oxidation states being much more stable than the last one. The activation of the Ru(III) complex, which occurs after the reduction of the metal ion to +2 state in the hypoxic environment characteristic of tumor cells has put the focus of the research on examining the anticancer potential of the Ru(II) complexes. Numerous studies have been published that focus on the properties and activities of these compounds with the emphasis placed on two main classes: organometallic Ru(II)-arene complexes and Ru(II)-polypyridine complexes [6]. The development of the so-called “half sandwich” Ru(II)-arene compounds (so-called “piano-stool” complexes) of the type [(η^6^-arene)Ru(YZ)(X)], where YZ is a bidentate chelating ligand and X is a leaving group, represents a significant advance in the development of structurally different ruthenium compounds with potential anticancer properties [4,8] (Figure 1). These compounds possess the broad possibilities of derivatization of the arene part that stabilizes the Ru(II) oxidation state, while the three remaining coordination sites, X, Y, and Z, can be functionalized with different coordination groups of different monodentate/chelate ligands, introducing thus the numerous biologically active compounds. As the solubility of these complexes in water is a problem for eventual clinical trials of these and similar compounds, nowadays research is focused on the synthesis of new, predominantly ionic complexes, with an increased number of halide ligands groups [9,10]. Today, some Ru(III) complexes have entered clinical trials, NAMI-A (imidazolium *trans*-[tetrachloro(dimethyl sulfoxide)(1H-imidazole)ruthenate(III)]), KP1019 (indazolium *trans*-[tetrachlorobis(1H-indazole)ruthenate(III)]) and the sodium salt analogue of KP1019, NKP-1339 [1].

Hydrazine has been a widely applied ligand in organometallic chemistry for a long time and several ruthenium compounds have been isolated and structurally characterized with bridging hydrazine moieties [11,12,13,14,15,16]. Closely related are rhodocenes, for instance, and ruthenium complexes with pyrazolato ligands [15,17,18,19]. Also, diazine ligands can connect two ruthenium atoms via both nitrogen donor atoms [20].

The research presented in this paper describes the synthesis and structural (IR, NMR, elemental analysis) of two Ru complexes with 1-naphtylhydrazyl ligand, [{RuCl(η^6^-*p*-cymene)}_2_(μ-Cl)(μ-1-*N*,*N′*-naphthyl)]Cl (**1**) and [{RuCl(η^6^-*p*-cymene)}_2_(μ-Cl)(μ-1-*N*,*N′*-naphthyl)]PF_6_ (**2**). The crystallographic structure of **2·2MeOH** was solved by X-ray crystallography. The cationic part [{RuCl(η^6^-*p*-cymene)}_2_(μ-Cl)(μ-1-*N*,*N′*-naphthyl)]+ (**2a**), that is common for both **1** and **2,** was extracted from the crystal structure and further used for the theoretical analysis which included density functional theory, natural bond orbital, and quantum theory of atoms in molecules analysis. The binding properties of **1** toward bovine serum albumin were examined by spectrofluorometry and molecular docking. The radical scavenging activity of the same compound was assessed towards DPPH^•^ and HO^•^ radicals. The cytotoxicity evaluation of **1** towards prostate PC-3 and colon HT-29 cells was performed. This compound was selected due to the higher solubility and acceptability of chloride ions over PF_6_^−^ in biological systems.

## 2. Results and Discussion

### 2.1. Synthetic Procedure and Crystallographic Structure

**Scheme 1:**
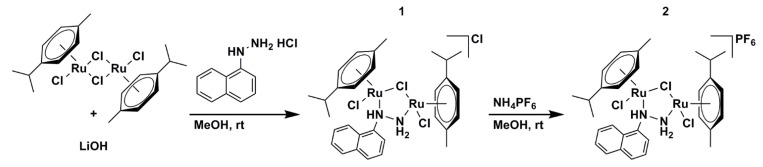
The synthetic route for compounds **1** and **2**.

Synthesis of the [{RuCl(η^6^-*p*-cymene)}_2_(μ-Cl)(μ-1-*N*,*N′*-naphthyl)]X was found to be successful only in methanol or 2-propanol, for X = Cl (**1**) or PF_6_ (**2**), respectively (Scheme 1). The interesting binding mode presented in this study is found also in the literature, but until now more steric hindering aromatic hydrazines were unknown for “piano-stool” Ru(II) complexes. Reports and studies include bridging hydrazine for ruthenium complexes in both dominant oxidation states (+2, +3) [11]. Examples of Ru(η^6^-arene) with bridging hydrazine are rare and substituted hydrazines are not well studied in this context [14], but phenylhydrazine was found to be a good bridging ligand in studies aiming for a better understanding of the dinitrogen reduction with ruthenium pincer complexes as models [12]. Cyclopentadienyl and indenyl ligands instead of arenes were also investigated with hydrazine [17]. Also in combination with other bridging units such as disulfide complexes were isolated and structurally characterized and are further examples of the versatile binuclear complex coordination with ruthenium [13,21]. Rigby et al., presented the general binding mode found for the title compound with rhodocene instead Ru(η^6^-*p*-cymene) to be unstable in solution and equilibrium in a wide temperature range. In this study, the same coordination of two Ru ions by hydrazine and one chlorido ligand was found with hints of existing equilibria in the solution [19]. The chemistry of Ru(II)(diene) was studied intensively with hydrazine ligand and closely related structural features were found in [16]. With excess ligand equivalents, the hydrazines are coordinated end-on to Ru(II), but lower amounts yield unsaturated coordination, and binuclear complexes are found. Structural characterization was possible by bridging chlorido, hydrido, and hydrazine ligands in the same complex [15]. The same five-membered ring of two ruthenium ions with one chlorine and two nitrogen atoms was found for pyrazolato-bridged complexes of ruthenium and rhodium [18]. All the former work in the field is giving good proof for the binding modes of hydrazine and the flexible coordination of ruthenium to them. The dependency on the reaction conditions and especially the equivalents of reactants as well as the solvent is offering a switch between bridging hydrazine and monodentate coordination. Therefore, coordination modes and properties can be manipulated during reactions. The bridging hydrazine together with chlorine and hydrogen bonding results in a bio-reactive complex in biological environment and other ligands can be bonded to one or both ruthenium ions.

Chemical shifts from the ^1^H and ^13^C NMR spectra agree with the expected resonances of the prepared complexes (Figures S1–S4). The IR spectra strongly suggest hydrogen bonding (*vide infra*) in the crystal structure which is stabilizing the bridging mode of 1-naphthylhydrazine as shown later.

**Figure 2:**
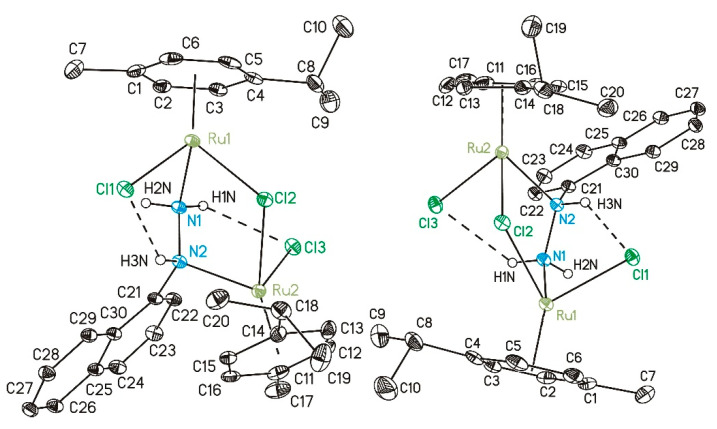
ORTEP (30% probability ellipsoids) of the molecular structure of [{RuCl(η^6^-*p-*cymene)}_2_(μ-Cl)(μ-1-*N*, *N*′-naphthyl)]^+^; and (**2a**) in two different perspective views. Carbon-bonded hydrogen atoms are omitted for clarity. Dashed lines refer to intramolecular N−H^…^Cl hydrogen bridges.

The X-ray structure was solved for **2·2MeOH** and the crystallographic parameters are presented in Table 1, while the bond lengths and angles are enlisted in Tables S1 and S2. The crystallographic structure of **2a** is shown in Figure 2. The crystallization process was performed in methanol. The compound crystallizes in the monoclinic space group and *I_2_/a* crystal system. These crystals consisted of binuclear Ru units with pseudo-octahedral geometry around metal atoms. Two Ru(II) atoms were bridged by hydrazine moiety in which two nitrogen atoms were connected to different Ru(II). Additionally, one chlorido ligand acted as a bridge between two Ru atoms. The coordination sphere included two *p*-cymene molecules and two terminal chlorido ligands. This is a half-sandwich complex in which the arene ring occupies three facial sites [22] through π-bonding. The Ru−C bond distances are between 2.161(3) and 2.216(3) Å, similar to the complexes bearing hexamethylbenzene moiety [22]. The bond distances Ru–Cl are in the range between 2.4063(7) and 2.4287(7) Å which is comparable to similar systems [22,23]. The distances between Ru and N are slightly different, (r(Ru−NH) = 2.193(2) Å and r(Ru−NH_2_) = 2.138(2) Å) due to the inclusion of the lone pair on the nitrogen of NH group into the delocalized system and proximity of aromatic rings. The outer sphere contains two methanol molecules and a counter ion, PF_6_^−^.

**Figure 3:**
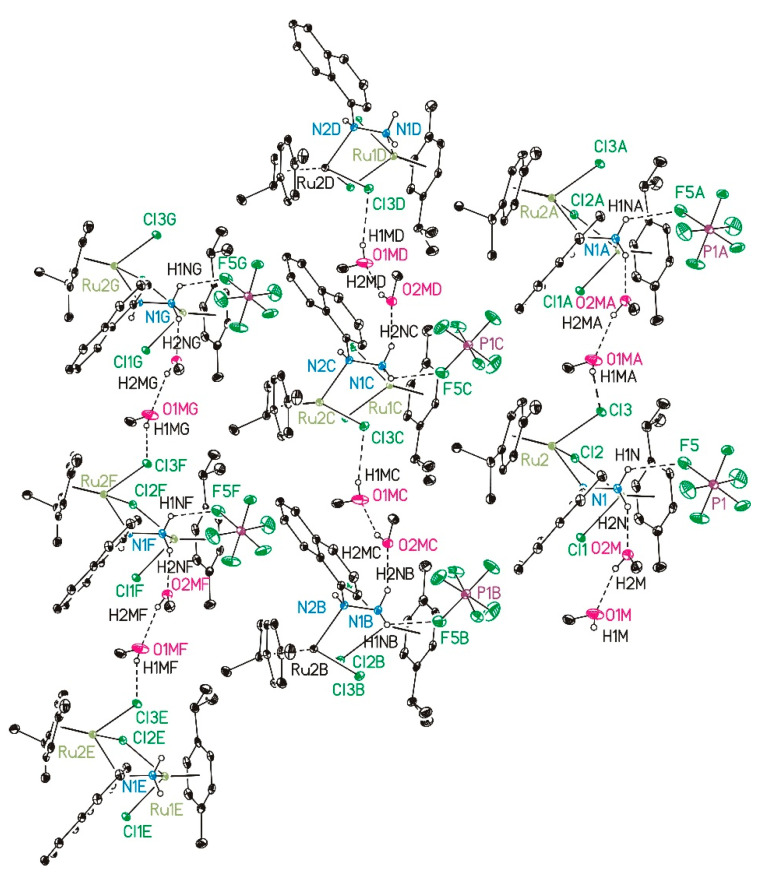
ORTEP (20% probability ellipsoids) of selected parts of three adjacent chains of **2·2MeOH** formed in the solid state due to the formation of hydrogen bridges along the crystallographic *b* axes. All carbon-bonded hydrogen atoms are omitted for clarity. Dashed lines refer to hydrogen bridges. Symmetry codes: “A” = x, y + 1, z. “B” = *x* + ½, −*y*, *z*. “C” = *x* + ½, −*y* + 1, *z*. “D” = *x* + ½, −*y* + 2, *z*. “E” = *x* + 1, −1 + *y*, *z*. “F” = *x* + 1, *y*, *z*. “G” = *x* + 1, *y* + 1, *z*.

In the solid-state **2·2MeOH** forms classical hydrogen bridges of two different types (Table 2). Intramolecular ones within **2a** (cf. Figure 2) are formed between the amino group and chlorido ligands (3.073 and 3.144 Å). These bonds deviate from linearity because of the steric hindrance of other ligands (138 and 126°). Intermolecular hydrogen bonds exist between two methanol molecules and between methanol molecules and an amino group. These interactions prove the importance of solvent molecules for the overall stability of the crystal. An additional intermolecular hydrogen bond is found between the amino group and the counter ion in the outer sphere, PF_6_^−^, as depicted exemplarily in Figure 3. Thereby, the chains are propagating along the crystallographic *b* axes (Figure 2). Non-classical interactions in the solid state are described next with the help of a Hirshfeld surface analysis.

### 2.2. Hirshfeld Surface Analysis

**Figure 4:**
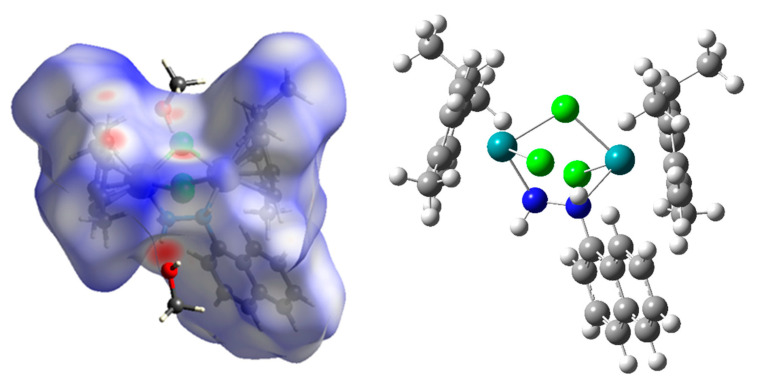
The Hirshfeld surface (**left**) and optimized structure (**right**) of **2a** (the following colors represent atoms white-hydrogen, grey-carbon, blue-nitrogen, green-chlorine, teal-ruthenium).

The intermolecular interactions govern the overall stability of the crystal structure of **2·2MeOH**. The fingerprint plots of different contact atoms are presented in Supplementary information as Figure S6, while the Hirshfeld surface of a single unit is shown in Figure 4. Within the structure, the ligands surround both metal atoms, therefore it is not expected to have interactions between Ru(II) of one unit and ligands or solvent molecules from another. The investigated compound does not have a significant number of polar groups in the structure of ligands, which limits the possible hydrogen bonds and other dipole-dipole interactions between ligands and solvent molecules. The surface in Figure 4 points out the positions (red spots) of the formed interactions with surrounding units, as presented in Figure S7. Thus, indicating the importance of intermolecular hydrogen bonds for network building outlined in the previous section. The highest number of contacts can be represented as H∙∙∙H, with a relative contribution of 59.7%. This result is expected as both *p*-cymene and naphtylhydrazine moieties are hydrogen-rich. These weak interactions are most important for the overall stability of crystal structure. Also, interactions between carbon and hydrogen atoms, C∙∙∙H account for 12.2% of all contacts. These contacts include weak interactions between a partially positive hydrogen atom and a π-electron cloud of aromatic moieties. A significant number of interactions include electrostatic interactions between hydrogen and chlorine atoms (H∙∙∙Cl, 8.8%), which are abundant in structure. These interactions can be classified as hydrogen bonds of the type C−H∙∙∙Cl and N−H∙∙∙Cl. The hydrogen bonds denoted as H∙∙∙F (C−H∙∙∙F and N−H∙∙∙F) account for 15.1%. Additional stabilization interactions within the structure are formed between hydrogen atoms and solvent molecules (methanol). These hydrogen bonds, H∙∙∙O, represent 2.3% of all interactions. The rest of the contacts, such as C∙∙∙C, C∙∙∙O, and C∙∙∙F are present below 1.5%.

### 2.3. Optimization of Structure, NBO, and QTAIM Analyses

The optimization of the structure was performed based on the crystallographic structure of **2a** at the B3LYP/6-31+G(d,p) level of theory for H, C, N, and Cl atoms and B3LYP/LanL2DZ level of theory for Ru atoms, as mentioned in the Materials and Methods section. The same level of theory was previously successfully used for the optimization of different Ru complexes [23,24,25]. The applicability of the chosen level of theory was assessed by the comparison of the experimental and theoretical bond lengths and angles. The parameters for comparison were the correlation coefficient (R) and mean absolute error (MAE). The MAE value was calculated as the average value of the absolute difference between experimental and theoretical lengths and angles. Tables S1 and S2 list these values along with the parameters R and MAE. The optimized structure, proven by the absence of imaginary frequencies as minima on the potential energy surface, is shown in Figure 4.

The R and MAE values for bond lengths are 0.999 and 0.031 Å (Table S1), while the same parameters for bond angles are 0.994 and 1.66° (Table S2), respectively. These values prove that the theoretical values reproduce well the crystallographic structure and that the selected level of theory is applicable to both compounds **1** and **2**, as the counter ion and methanol molecules were removed during the optimization. Although almost identical to the crystallographic one, an optimized structure was necessary for further theoretical considerations. The obtained complex contains *p*-cymene and naphthyl moieties with elongated delocalization and the rotation of their bonds is limited, therefore it was expected to have a high correlation coefficient and low values of MAE. In the crystal structure of **2a**, the Ru−Cl bond lengths are 2.4063(7)/2.4172(7) (Ru1) and 2.4287(7)/2.4150(7) Å (Ru2), while in theoretical structure these bonds are elongated for 0.06 Å. The Ru−N bond lengths are 2.138(2)/2.193(2) and 2.189/2.238 Å in experimental/theoretical structure. These elongations are a consequence of a system relaxation, which occurs during the optimization process because the optimizations are performed for the isolated compounds in a vacuum. In the crystal structure, there are additional interactions that stabilize the overall structure, as shown in the previous sections. The same applies to bond angles. The experimental bond angles Cl−Ru−Cl are 86.19(2) and 85.65(2)°, while theoretical bond angles are 88.08 and 88.00°. The experimental bond angles N−Ru−Cl are also well-reproduced with an increase of 0.2°. The hydrazyl moiety forms an angle with both Ru ions of 115.98(15)°. The stability of the compound was additionally investigated by the NBO and QTAIM analyses to quantify the interactions’ strength.

As shown in Figure 2, the structure of the title compound consists of several units bound through different interactions. Therefore, it is of utmost interest to analyze the stabilization interactions within all of these units. The strongest stabilization interactions within *p*-cymene moiety are formed between C−C bonds, denoted as π(C−C) → π*(C−C), with energies between 30.6 and 33.3 kJ mol^−1^. The same type of interactions can be found in the napthtylhydrazine moiety, although these interactions are more numerous and characterized by larger energies due to the extended delocalization through coupled aromatic rings. These stabilization interactions have energies between 62.6 and 78.9 kJ mol^−1^. The presence of heteroatoms in the hydrazine group increases the number of possible interactions. The lone pair on nitrogen in position N2 is delocalized over double bonds through LP(N) → π*(C−C) interaction with an energy of 51.5 kJ mol^−1^. There is an additional stabilization, denoted as LP(N) → σ*(N−C) with an energy of 22.3 kJ mol^−1^. The extended delocalization of napthtylhydrazine moiety is expected and significantly increases the overall stability. The interactions between donor atoms and Ru(II) are also important factors. The interaction between the π-electron cloud and Ru atom is formed through interactions denoted as π(C−C) → LP*(Ru) and it includes all of the carbon-carbon pairs of the aromatic core. The strength of these interactions is around 470 kJ mol^−1^, which proves that these aromatic structures form very stable compounds with Ru atoms. There are other interactions of the same type with energies of 60 kJ mol^−1^. Nitrogen atoms of hydrazine moiety also interact with Ru(II), through interactions that have an energy of 370 kJ mol^−1^. The stable compounds formed between the nitrogen atom and Ru have previously been reported in the literature [26,27], which is experimental proof of the calculated stabilization interactions. Weaker interactions, σ(N-H) → LP*(Ru) with stabilization energy of 50 kJ mol^−1^ also exist in the structure. The chloride ligands act as electron donors through lone pairs. The interactions between different lone pairs of chlorine and Ru atoms, LP(Cl) → LP*(Ru) have an energy of 350 kJ mol^−1^, along with several other weaker interactions. Weaker interactions between chlorine and Ru atoms are formed in the case of bridging chlorido ligand, 240 kJ mol^−1^. Chlorine atoms additionally interact with the NH group of hydrazine moiety through weak hydrogen bonds with an energy of 50 kJ mol^−1^, as previously shown by the crystallographic structure (Figure 2) and Hirshfeld analysis (Figure 4). It is important to notice that all of the donors, namely chlorine, nitrogen, and aromatic moiety, interact with Ru(II) through stabilization interactions of similar strength.

**Figure 5:**
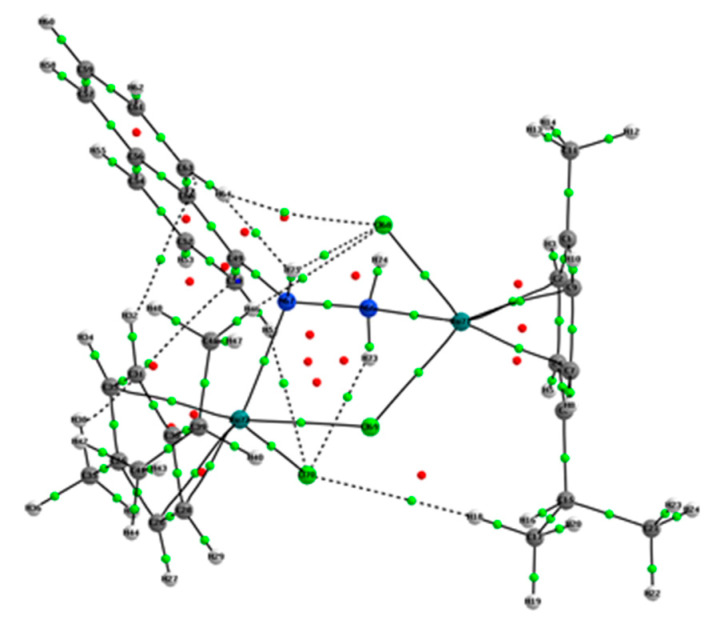
Molecular graph of **2a**, dashed lines indicate bond paths, small green, and red points refer to the bond critical points and ring critical points.

QTAIM has gained widespread acceptance for the investigation of various interactions in organometallic compounds through calculations of electron density and its Laplacian [28]. Different metal–metal and metal–ligand bonds have been subjected to this type of analysis [29,30,31]. Within this approach, a molecular graph defines the positions of critical points and paths that connect bonded atoms, both of which are an indicator of the chemical bond’s existence [32]. The molecular graph of **2a** is shown in Figure 5 with BCPs denoted as green dots and RCPs as red dots. The strongest bonds within the investigated structure are C=C with electron density around 0.3 au and Laplacian between −0.75 and −0.88 au. These values are higher in the case of naphthyl moiety due to the extended delocalization through two adjacent ring structures. Single carbon-carbon bonds have lower values of electron density (~0.25 au) and Laplacian (between −0.63 and −0.60 au). These parameters also indicate that the C−C bond between the aromatic ring and methyl group is stronger than the same bond between the aromatic ring and isopropyl group, which is consistent with the previously discussed NBO results due to the hyperconjugation effect. The resonance effect between hydrazyl and naphthyl moieties leads to the formation of a strong C−N bond with an electron density of 0.27 au and Laplacian of −0.79 au. The N−N bond is partially weakened by the formation of interaction between N and Ru, although this bond is still strong with an electron density of 0.29 au. The bonds between carbon/nitrogen and hydrogen atoms are the weakest of all covalent bonds. All the mentioned bonds fulfill the previously presented criteria for the closed-shell interactions. The interactions between carbon atoms of *p*-cymene moiety and Ru are characterized by the electron density of 0.07 au and Laplacian of 0.21 au, as an example of open-shell interactions. Similar values of the BCP’s parameters were obtained in the case of a half-sandwich Ru (II) complex containing β-diketiminate ligand [33]. The interaction between Cl and Ru has Laplacian equal to 0.18 au and electron density equal to 0.06 au, which makes these interactions weaker than those previously discussed. The interaction between the nitrogen atom (−NH_2_) and Ru ion has an electron density of 0.08 au and Laplacian of 0.33 au. The interaction with the nitrogen atom (−NH−) is weaker, with lower values of electron density and Laplacian. Other than this, all other interactions between Ru(II) and donor atoms have almost equal strength.

### 2.4. Experimental and Theoretical IR and NMR Spectra

**Figure 6:**
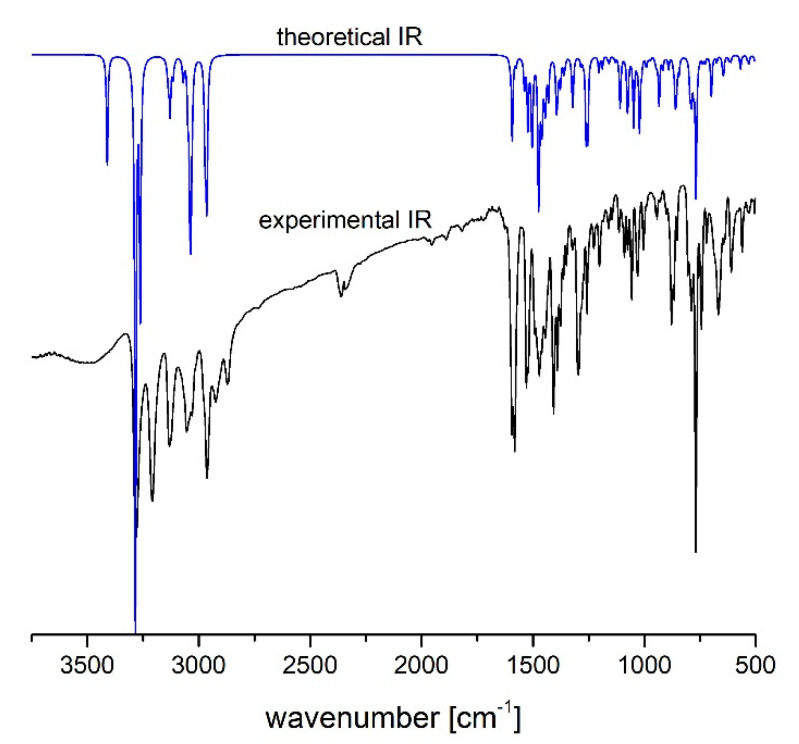
The experimental IR spectrum of **1** and theoretical IR spectrum of **2a**.

The experimental IR spectrum of **1** and theoretical IR spectrum of **2a** are given in Figure 6. The experimental spectra of **1** and **2** are shown in Figure S5 and Figure 6 and they show great resemblance, except for the bands belonging to the counterions. The theoretical spectrum was predicted based on the optimized structure as additional proof that the selected level of theory was appropriate for the description of the structure. The obtained theoretical wavenumbers were systematically overestimated, which required the use of the factor of 0.974. The corrected wavenumbers are discussed in the following text. The correction factor was used to include the differences in optimization of an isolated compound in vacuum and experimental procedure which included the powder in the KBr pellet. The importance of intermolecular interactions for the stability of solid-state structures has been previously discussed.

In the range between 3500 and 2800 cm^−1^ several prominent bands can be observed. In the experimental spectrum, there is a doublet due to the presence of the primary amino group (N−H stretching vibration) at 3277 and 3200 cm^−1^. In the theoretical spectrum, these bands are represented by intense bands at 3285 and 3265 cm^−1^. The difference of several cm^−1^ is due to the intermolecular interactions formed by the polar groups with surrounding species. The C−H stretching vibrations of aromatic moiety are located at 3120 and 3034 cm^−1^ in the experimental spectrum and 3129 cm^−1^ in the theoretical spectrum. The vibrations have somewhat higher wavenumbers, probably due to the coordination of aromatic rings to the Ru(II) [34]. The C−H stretching vibrations of sp^3^ carbon atoms lead to the appearance of two distinct bands at 2956 and 2916 cm^−1^. The first one belongs to the methyl group of a *p*-cymene moiety, while the second represents vibrations of the isopropyl group of the same moiety. The theoretical positions of these bands are 3042 and 2963 cm^−1^. These two groups have distinct wavenumbers due to the increased hyperconjugation effect of the aromatic ring to the methyl group.

The second part of the spectrum, below 1598 cm^−1^, includes several medium to high-intensity bands that can be assigned to the mixed vibration modes. The band at 1598 and 1320 cm^−1^ represent the deformation vibration of the primary amino group. Several medium bands between 1528 and 1000 cm^−1^ are due to the bending vibrations including the aromatic rings of *p*-cymene and naphthyl moieties. The N−Ru vibration can be observed at 466 cm^−1^. The torsional and rocking vibrations of ligands are positioned below 500 cm^−1^, as previously shown in references [35,36]. Due to the experimental conditions and weak interaction, the Cl−Ru vibrations can only be observed in the theoretical spectrum at 280 cm^−1^, which is in accordance with the paper by Durig that describes trichlorotriaquoruthenium(III) [37]. The position of the Cl−Ru absorption band can be used to distinguish between bridging and terminal chlorido ligands in structure [38].

NMR spectra of compounds **1** and **2** were recorded in MeOD which was followed in the theoretical analysis for the prediction of this type of spectra of **2a**. The experimental spectra of both compounds are presented in Figures S1–S4. The theoretical chemical shifts were calculated relative to TMS optimized at the same level of theory. The experimental and theoretical chemical shifts are presented in Table S3 and the correlation coefficient and MAE were obtained to verify the applicability of the chosen level of theory.

The ^1^H NMR chemical shifts show a high correlation between experimental and theoretical values (0.994) with an MAE of 0.24 pm. The lowest values of chemical shifts were obtained for the isopropyl methyl groups between 1.05 and 1.34 ppm in the experimental and 1.20 and 1.47 ppm in the theoretical spectrum. The methyl groups of *p*-cymene, represented by singlets, are located at 1.88 and 2.15/1.73 and 1.95 ppm in the experimental/theoretical spectrum. The heptets belonging to the CH group of isopropyl moieties show a difference of 0.2 ppm between experimental and theoretical spectra. The mentioned groups are not influenced significantly by the complex compound formation. It is also important to mention that two *p*-cymene moieties are not identical chemical environments which leads to different values of chemical shifts. The proximity of electronegative nitrogen atoms leads to the shift in the position of hydrogen atoms (8.65 ppm for H1 and 7.8 ppm for H3). The rest of the hydrogen atoms have their usual positions. In the case of ^13^C NMR spectra, a high correlation coefficient (0.996) and low MAE (3.54 ppm) also prove the representation of the crystallographic structure. The methyl group carbon atoms of isopropyl moiety have chemical shifts between 18.35 and 22.75 ppm in the experimental spectrum, and between 18.13 and 27.30 ppm in the theoretical spectrum. These values were overestimated due to the calculations on isolated molecules without explicit solvent effect which might induce the conformational changes that influence the interactions with surrounding groups. A difference of several ppm was also calculated for methyl groups of *p*-cymene. The rest of the values were well-reproduced, which is expected due to the stability of structures and extended delocalization, which prevents conformational changes as a consequence of the solvent effect. The highest value of chemical shift was calculated for the carbon atom adjacent to the hydrazine group (147.70 ppm in the experimental and 143.60 ppm in the theoretical spectrum). Other theoretical studies point out that PBE0 functional had superior performance in calculating Ru compounds’ NMR chemical shifts [39,40,41,42]. The calculated chemical shifts of **2a** at PBE0/6-31+G(d,p)(H,C,N,Cl)/LanL2DZ(Ru) and PBE0/6-311++G(d,p)(H,C,N,Cl)/LanL2DZ(Ru) levels of theory are presented in Table S4. Two basis sets for the non-metals were used to investigate the effect of basis set size on the obtained results. In the case of ^1^H NMR chemical shifts the statistical parameters show that both levels of theory perform similarly to the previously used one (R ≈ 0.994, MAE ≈ 0.23 ppm). On the other hand, the differences are more noticeable for ^13^C NMR shifts. When functional is changed from B3LYP to PBE0, the R coefficient stay the same, but the MAE is lowered to 3.08 ppm. A further change of basis set leads to the lower MAE value of 3.05 ppm. These results prove that PBE0 performs slightly better than B3LYP in the case of studied Ru compounds, although more detailed studies are needed.

### 2.5. Spectrofluorometric Measurements and Molecular Docking with BSA

**Figure 7:**
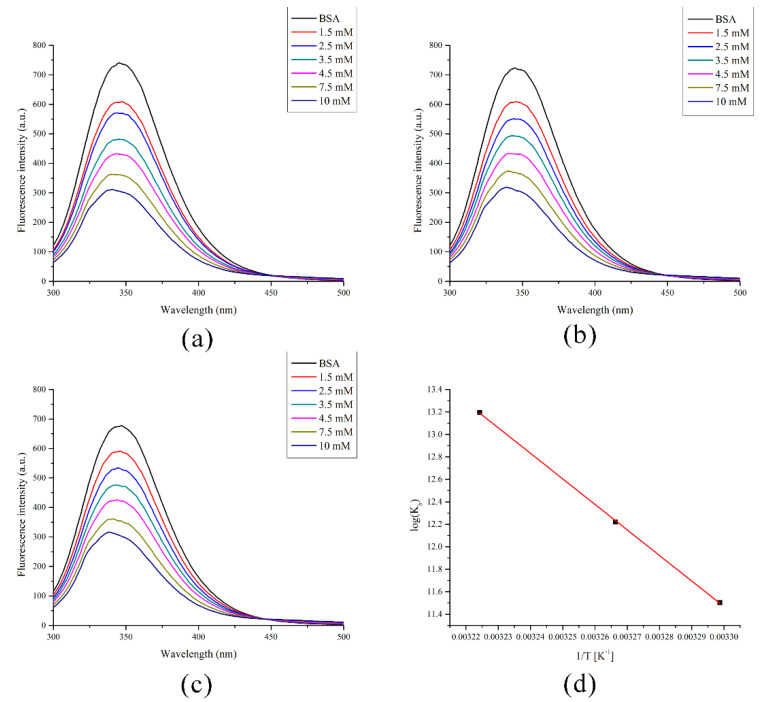
The fluorescence spectra of BSA after the addition of various amounts of **1** at (**a**) 30 °C; (**b**) 33 °C; (**c**) 37 °C; and (**d**) Van’t Hoff diagram for the binding process of **1** and BSA.

The binding of **1** to BSA, as an important transport protein, was investigated by spectrofluorimetric titration. When BSA is irradiated by the excitation wavelength of 280 nm, the intrinsic fluorescence of tryptophan and tyrosine is activated. The fluorescence emission at 345 nm is mostly due to the tryptophan residues in positions 134 and 212 and it is dependent on the chemical environment of these two amino acids. The change in the secondary structure of BSA leads to a decrease in fluorescence intensity. The fluorescent spectra of BSA before and after various additions of **1** are shown in Figure 7 for three temperatures (30, 33, and 37 °C). The addition of **1** led to a decrease in the fluorescence intensity in a concentration-dependent manner, as presented. The binding constants were determined from the double log Stern-Volmer plots (Figure S8) as the dependence of fluorescence intensity on the concentration of **1**. This analysis allowed the determination of the number of **1** bound to one BSA molecule. For all three temperatures, this number is close to 1 indicating a 1:1 binding ratio similar to the examples in the literature [34]. The binding constants were 9.90 × 10^4^, 2.03 × 10^5^, and 5.37 × 10^5^ M^−1^ for the measurements at 30, 33, and 37 °C, respectively. These values indicate strong binding of **1** to BSA, comparable to that for ibuprofen (3.6 × 10^6^ M^−1^) and diazepam (1.6 × 10^6^ M^−1^), which are known to bind albumin tightly [43,44]. The binding constant of **1** at normal body temperature is also comparable to those of different Ru-arene complexes found in the literature, for example, various cyclopentadienylruthenium(II) compounds of acetophenone-4(N)-substituted thiosemicarbazones (1.03–7.77 × 10^5^ M^−1^), half-sandwich Ru(II) arene chloride-complexes of quinoline substituted benzo[d]imidazole, benzo[d]oxazole and benzo[d]thiazole (3.63–6.52 × 10^5^ M^−1^), and [Ru(HL)(CH_3_CN)(CO)(PPh_3_)_2_] with HL = 4-oxo-4H-pyran-2,6-dicarboxylic acid (2.89 × 10^6^ M^−1^) [45,46,47]. The binding constants at various temperatures were further used for the determination of the thermodynamic parameters of the binding process (Figure 7d) that are presented in Table 3.

The change in enthalpy of binding was calculated to be 189 kJ mol^−1^, while the change in entropy of binding was 719 J mol^−1^ K^−1^. The change in Gibbs free energy of binding was in the range between −29 and −34 kJ mol^−1^, which proved the spontaneity of the binding process. When the values of thermodynamic parameters are compared and their contributions to the change in Gibbs free energy of binding determined, it can be concluded that the binding process is driven by the hydrophobic effect. It can be assumed that during the binding there is a decrease in the order of the system and probably disintegration of the structure of **1**. Molecular docking studies were performed to examine the possible interactions between **1** and active pockets of BSA.

The molecular docking studies were performed for **2a** and naproxen in the binding pocket of ligand. The native bound ligand (naproxen) was extracted from BSA, and binding pocket analysis was performed. After that, re-docking was performed with the investigated compounds to generate the same docking pose as found in its co-crystallized form. This step was performed to compare the theoretical binding affinity of **2a** with the reference drug, naproxen (NPS) [48], and correlate it with the experimental inhibition constant.

**Figure 8:**
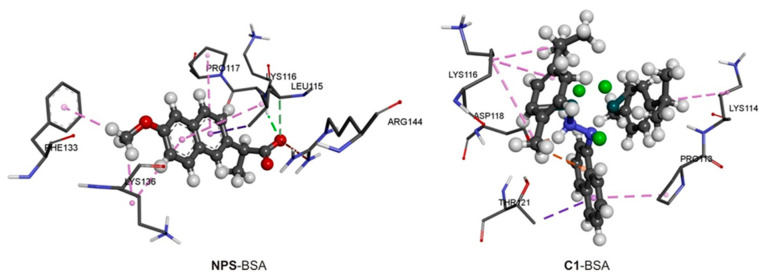
The hydrogen bond (green and orange dotted lines) and hydrophobic (rose pink dotted lines) docking interactions of the most stable conformations of NPS and **1** with BSA.

The most stable docking conformations of **2a** and NPS are presented in Figure 8 and Table 4. A more negative value of free energy of binding (ΔG_bind_) indicates better inhibition. The inhibitory activities of compounds **1** and NPS towards BSA were ranked based on their lowest binding energy involved in the complex formation at the active sites.

As can be seen from Table 4, both compounds, **1** and **NPS**, bind strongly to BSA receptors. The docking analyses of investigated molecules revealed that several non-covalent interactions existed between investigated molecules and target receptors. The most prominent interactions are hydrogen bonds, π-anion, π-cation, and π-alkyl interactions (Figure 8). ASP in position 118 in the primary structure of the BSA protein chain has a predominant role as the active site of receptor regarding **2a**. This amino acid forms strong hydrogen bonds (bond lengths range from 1.73 to 2.05 Å), while ASP118, THR121, LYS116, LYS114, and PRO113 form weak π-anion, π-cation, and π-alkyl interactions with the benzene ring of investigated ligand (Figure 8). On the other hand, LYS116 in the primary structure of BSA forms hydrogen bonds with C=O groups of NPS. In addition, LYS116, LEU115, PRO117, PHE133, ARG144, and LYS136 form weak alkyl-π, and π-π interactions with the benzene ring of NPS. Naproxen, as the one NSAID, for the same receptor target, showed remarkably less negative binding energy (Δ*G*_bind_ = −21.3 kJ mol^−1^ to BSA) indicating that **1** has a higher affinity to the BSA receptor (Table 4), in comparison to NPS [48]. It is important to outline that the calculated binding energy of **2a** to BSA at normal body temperature is only 2 kJ mol^−1^ higher than the experimental one, proving that molecular docking study results are complementary to the spectrofluorimetric methods.

Based on the results presented in Table 4, it is clear that ligand efficiency is not determining factor for the value of the binding energy. On the other hand, the main contribution to the binding energy comes from the sum of the dispersion, repulsion, and hydrogen bond energies (Table 4). It should be noted that electrostatic interactions also significantly contribute to the stabilization of the complex with **2a** compared to NPS. The torsional energies are lower for NPS due to the smaller size and lower flexibility of this molecule in comparison to **2a**.

### 2.6. Radical Scavenging Activity

**Figure 9:**
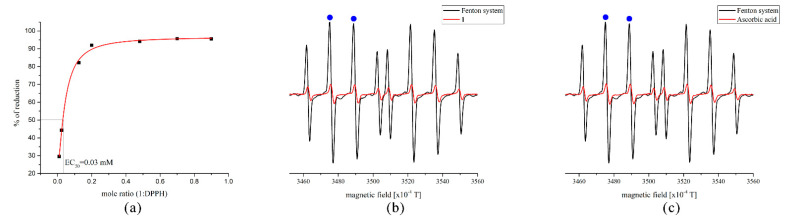
(**a**) Dependence of the reduction percentage of DPPH^•^ on the mole ratio between **1** and DPPH^•^; (**b**) EPR spectra of DEPMPO-HO^•^ adduct with and without 1; and (**c**) EPR spectra of DEPMPO-HO^•^ adduct with and without ascorbic acid. (Blue circles denote peaks used for evaluation of antiradical capacity).

The evaluation of radical scavenging activity was performed towards two radicals, namely DPPH^•^ and HO^•^. The first radical is a standard long-living species used as a preliminary test for the overall activity, although there are several limitations, which primarily include the size of the antioxidant that neutralizes this specie [49]. Figure 9 shows the curve for the determination of the EC_50_ value of **1**, which was determined to be 30 μM under the presented experimental conditions. This value is comparable to the standard antioxidants such as kaempferol, quercetin, ascorbic acid, Trolox, and (+)-α-tocopherol [50]. It should be kept in mind that DPPH^•^ is usually reduced by the hydrogen atom abstraction from antioxidant or electron transfer from phenoxide anion as shown in references [51,52,53]. It can be postulated that the electron transfer from the naphthylhydrazine moiety is partially responsible for the DPPH^•^ scavenging, as **1** does not contain any of the common hydrogen atom donating groups [54]. The reactivity of **1** and ascorbic acid towards HO^•^, generated in the Fenton system, was examined by the EPR spectroscopy. Figure 9 shows the spectra of DEPMPO-HO^•^ adduct with and without mentioned compounds. The low-filed EPR signals used for the calculation of the scavenging activities are marked with blue points. The scavenging activities of 0.5 μM solutions of **1** and ascorbic acid are 86.2 and 85.2%, respectively. Based on these results, it can be concluded that the antiradical activity of **1** is comparable to the activity of ascorbic acid. Various mechanisms have been proposed for the reaction between ascorbic acid and HO^•^, examined both theoretically and experimentally [55,56], and radical adduct formation has been outlined as one of the thermodynamically and kinetically preferred mechanisms. It is believed that this mechanism of reduction applies to **1** due to the multitude of double bonds, which are usual positions for radical adduct formation [57]. This mechanism also explains the higher reactivity of 1 towards HO^•^ than DPPH^•^, although further theoretical studies are required.

### 2.7. Cytotoxic Activity

**Figure 10:**
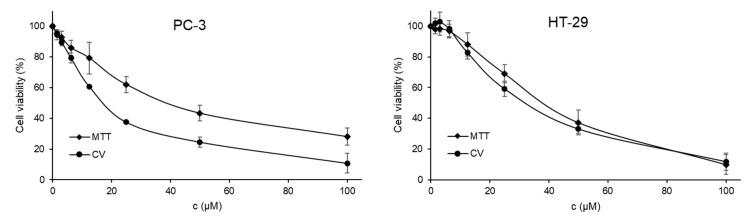
Dose-dependent response of PC-3 and HT-29 cells treated with **1**. IC_50_ [µM] on: PC-3 40.52 ± 3.89 (MTT), 16.92 ± 1.42 (CV); HT-29 37.90 ± 2.33 (MTT), 11.49 ± 1.87 (CV).

MTT and CV assays were employed to determine the cytotoxic potential of **1**. Prostate PC-3 and colon HT-29 cells were treated with different concentrations of the title compound for 48 h (Figure 10). The cytotoxic potential, IC_50_ concentrations (µM, see Figure 10 caption), relieved that MTT and CV assays are not in agreement. The higher IC_50_ concentrations obtained with MTT, in comparison to CV, indicate unquestionably that **1** slows down the respiration of both PC-3 as well as HT-29 cells, pointing out that the mitochondria function is disrupted with the action of Ru(II) binuclear complex. Taking into consideration the CV assay, the prepared compound showed slightly higher activity against the HT-29 cells than against the PC-3 cell line. In direct comparison with cisplatin (IC_50_ [µM] on PC-3 10.66 ± 0.49 (MTT), 9.26 ± 3.03 (CV); HT-29 5.03 ± 0.52 (MTT), 0.92 ± 0.33 (CV)), **1** showed lower activity against selected cell lines [58]. Even though the binuclear Ru(II) complex is less active than cisplatin, the gold standard in cancer treatment, higher tolerance of relatively high ruthenium amounts in biological systems might likely indicate potential applicability for cancer treatment and the mechanism of action will be the subject of a forthcoming study. Therefore, further biological studies are required to estimate the real potential of **1** toward different cancer cell lines.

## 3. Materials and Methods

### 3.1. Synthesis

All synthetic work was carried out in an appropriate atmosphere (N_2_) using standard Schlenk techniques. All chemicals and solvents were commercially obtained and used as received. [{RuCl_2_(η^6^-*p*-cymene)}_2_] and 1-naphthylhydrazine hydrochloride were purchased from TCI. Solvents were dried with respective molecular sieves at least 2 weeks before use. Elemental analyses were performed by the Microanalytical Laboratory of the University Leipzig (Heraeus VARIO EL oven).

#### 3.1.1. Synthesis of [{RuCl(η^6^-p-Cymene)}_2_(μ-Cl)(μ-1-N,N′-Naphthyl)]Cl (1)

In a 25 mL flask [{RuCl_2_(η^6^-*p*-cymene)}_2_] (64 mg, 0.1 mmol), LiOH × H_2_O (3.3 mg, 0.08 mmol), 1-naphthylhydrazine hydrochloride (20 mg, 0.1 mmol) and 2-propanol (5 mL) were added at room temperature. The mixture was vigorously stirred and meanwhile degassed by adding nitrogen through a syringe for 15 min. The orange reaction suspension started to clear up slowly and a fine precipitate formed right after. The reaction was run overnight, and diethyl ether (10 mL) was added to the orange suspension. After cooling the reaction mixture for 1 h at −20 °C, the yellow-orange product was collected via filtration, washed with diethyl ether (3 × 5 mL), and dried in air. Yield 52 mg, 68%.

^1^H NMR (400 MHz, MeOD) δ (ppm) 8.56 (dd, *J* = 8.6, 1.0, 1H, naph), 8.29 (dd, *J* = 7.7, 1.1, 1H, naph), 8.10 (d, *J* = 8.2, 1H, naph), 7.99 (d, *J* = 8.1, 1H, naph), 7.80 (ddd, *J* = 8.5, 6.9, 1.4, 1H, naph), 7.72–7.67 (m, 2H, naph), 5.68 (d, *J* = 6.8, 2H, cym-C*H*CH), 5.57–5.52 (m, 2H, cym-C*H*CH), 5.38 (d, *J* = 5.6, 1H, cym-C*H*CH), 5.26 (d, *J* = 5.6, 1H, cym-C*H*CH), 4.98 (d, *J* = 5.8, 1H, cym-C*H*CH), 4.26 (d, *J* = 5.4, 1H, cym-C*H*CH), 2.91 (hept, *J* = 7.0, 1H, cym-C*H*CH_3_), 2.66 (hept, *J* = 7.0, 1H, cym-C*H*CH_3_), 2.15 (s, 3H, cym-CC*H*_3_), 1.88 (s, 3H, cym-CC*H*_3_), 1.34 (d, *J* = 6.9, 3H, cym-CHC*H*_3_), 1.29 (d, *J* = 7.0, 3H, cym-CHC*H*_3_), 1.28 (d, *J* = 6.9, 3H, cym-CHC*H*_3_), 1.05 (d, *J* = 7.0, 3H, cym-CHC*H*_3_) (Figure S1). ^13^C NMR (101 MHz, MeOD) δ (ppm) 147.70, 135.21, 130.69, 129.80, 128.89, 128.35, 127.15, 125.27, 121.40, 121.16 (10× naph), 107.52 + 106.98 (2× cym-*C*CH*C*H_3_), 98.42 + 97.42 (2× cym-*C*CH_3_), 85.95 + 84.62 + 84.59 + 83.20 + 83.16 + 82.07 + 81.20 + 78.57 (8× cym-*C*HCH), 32.02 + 31.79 (2× cym-*C*HCH_3_), 22.75 + 22.71 + 22.07 + 21.15 (4× cym-CH*C*H_3_), 18.46 + 18.35 (2× cym-C*C*H_3_) (Figure S2). Anal. Calcd for C_30_H_38_Cl_4_N_2_Ru_2_ (770.59): C, 46.76; H, 4.97; N, 3.64. Found: C, 46.41; H, 4.80; N, 3.40.

#### 3.1.2. Synthesis of [{RuCl(η^6^-p-Cymene)}_2_(μ-Cl)(μ-1-N,N′-Naphthyl)]PF_6_ (2)

In a 50 mL flask [{RuCl_2_(η^6^-*p*-cymene)}_2_] (129 mg, 0.2 mmol) was dissolved in methanol (6 mL) at room temperature. The mixture was vigorously stirred and meanwhile degassed by adding nitrogen through a syringe for 5 min. LiOH × H_2_O (6.6 mg, 0.16 mmol) was added to the reaction mixture, and in the meantime (15 min) all starting material dissolved. After an additional 30 min, 1-naphthylhydrazine hydrochloride (40 mg, 0.2 mmol) was added in one portion and the obtained clear red solution was stirred overnight. Subsequently, NH_4_PF_6_ (10 eq, 330 mg, 2 mmol) was added and diluted with diethyl ether (10 mL) after 1 h. After another 24 h diethyl ether (5 mL) and n-hexane (20 mL) were added to the reaction mixture. The orange product, as precipitate, was filtered off, washed with diethyl ether (2 × 5 mL), and dried in air. Yield 171 mg, 97%.

^1^H NMR (400 MHz, MeOD) δ (ppm) 8.56 (d, *J* = 8.9, 1H, naph), 8.29 (dd, *J* = 7.7, 1.1, 1H, naph), 8.10 (dt, *J* = 8.2, 0.8, 1H, naph), 7.99 (d, *J* = 8.3, 1H, naph), 7.80 (ddd, *J* = 8.5, 6.9, 1.4, 1H, naph), 7.73–7.66 (m, 2H, naph), 5.68 (d, *J* = 6.6, 2H, cym-C*H*CH), 5.57–5.52 (m, 2H, cym-C*H*CH), 5.38 (d, *J* = 6.2, 1H, cym-C*H*CH), 5.26 (d, *J* = 6.0, 1H, cym-C*H*CH), 4.98 (d, *J* = 6.2, 1H, cym-C*H*CH), 4.27 (d, *J* = 6.0, 1H, cym-C*H*CH), 2.91 (hept, *J* = 7.0, 1H, cym-C*H*CH_3_), 2.67 (hept, *J* = 7.1, 1H, cym-C*H*CH_3_), 2.16 (s, 3H, cym-CC*H*_3_), 1.88 (s, 3H, cym-CC*H*_3_), 1.34 (d, *J* = 6.9, 3H, cym-CHC*H*_3_), 1.29 (d, *J* = 7.0, 3H, cym-CHC*H*_3_), 1.28 (d, *J* = 6.9, 3H, cym-CHC*H*_3_), 1.05 (d, *J* = 7.0, 3H, cym-CHC*H*_3_) (Figure S3). ^13^C NMR (101 MHz, MeOD) δ (ppm) 147.75, 135.22, 130.68, 129.78, 128.88, 128.33, 127.15, 125.31, 121.44, 121.21 (10× naph), 107.53 + 107.00 (2× cym-*C*CH*C*H_3_), 98.39 + 97.38 (2× cym-*C*CH_3_), 85.99 + 84.64 + 83.28 + 83.16 + 82.54 + 82.46 + 82.05 + 81.19 + 78.56 (8× cym-*C*HCH), 32.03 + 31.81 (2× cym-*C*HCH_3_), 22.77 + 22.72 + 22.07 + 21.16 (4× cym-CH*C*H_3_), 18.48 + 18.35 (2× cym-C*C*H_3_) (Figure S4). Anal. Calcd for C_30_H_38_Cl_3_F_6_N_2_PRu_2_ (880.10): C, 40.94; H, 4.35; N, 3.18. Found: C, 41.23; H, 4.18; N, 3.35.

### 3.2. X-ray Analysis

The crystallographic structure of **2·2MeOH** was determined based on the X-ray diffraction analysis of a single crystal on a Rigaku Oxford Gemini S diffractometer at 100 K using Mo-Kα radiation. Absorption corrections were made with the SCALE3 ABSPACK algorithm as implemented in the CrysAlisPro software [59]. Direct methods were used for solving the structure with SHELXS-2013 and refined by full-matrix least-square routines against F^2^ with SHELXL-2013. Non-hydrogen atoms were refined with anisotropic parameters, while hydrogen atoms bonded to the carbon atoms were placed in the calculated positions, according to the riding model. The positions of O- and N-bonded hydrogens were taken from difference Fourier maps and refined with appropriate constraints. CCDC-2217194 contains the supplementary crystallographic data for **2·2MeOH**. These data can be obtained free of charge from The Cambridge Crystallographic Data Centre via www.ccdc.cam.ac.uk/data_request/cif (accessed on 1 November 2022).

### 3.3. Spectroscopic Analysis

NMR spectra were measured on a Bruker AvanceTM 400 MHz Spectrometer (^1^H, 400.13 MHz; ^13^C, 100.50 MHz). Chemical shifts (δ) are given in parts per million (ppm) and coupling constants (J) in hertz (Hz). Referencing was done to internal tetramethylsilane or respective undeuterated solvent residue signals. The infrared spectra were recorded on a Thermo Nicolet—Avatar 370 FTIR spectrometer, Thermo Fisher, Waltham, MA, USA in the range between 4000 and 400 cm^−1^. The KBr pellet technique was used with the mass ratio **1**: KBr = 4 mg:150 mg.

### 3.4. Hirshfeld Surface Analysis

Interactions formed between fractions of the crystal structure are important for overall stability. Therefore, it is of utmost interest to quantify them and determine the most important contacts between fragments. The Hirshfeld analysis is based on the contacts between interacting atoms. The crystal structure of **2** was investigated in the Crystal Explorer [60]. Hirshfeld’s surface is represented by a graph showing two distances, one between the two nearest nuclei (d_e_) and the second one being the distance from nuclei to the external surface (d_i_) [61,62,63]. The normalized distance (d_norm_) is colored in red, white, and blue, if the shown distance is shorter, equal, or longer than the Van der Waals separation between atoms. The values of d_norm_ shown in this paper are between −0.4939 (red) and 1.1572 a.u. (blue). The fingerprint plots are prepared for each of the pairs of interacting atoms and they are shown in the Supplementary material.

### 3.5. Theoretical Calculations

The geometry of obtained compound was optimized in the Gaussian Program Package [64] starting from the crystallographic structure of **2** without any geometrical constraints. The authors have optimized the structure without methanol and counter ions, and therefore the same structure applies to both complexes. The global hybrid generalized gradient approximation (GAA) functional B3LYP [65] was employed in conjunction with 6-31+G(d,p) [66] basis set for H, C, N, and Cl atoms and LanL2DZ [67,68] basis set for Ru. The absence of the imaginary frequencies showed that the global minimum on the potential energy surface was found. The vibrational spectra were analyzed and viewed in the GausView program [64]. The conductor-like polarizable continuum model (CPCM) [69] was implemented for the optimization of structure in CH_3_OD, to encounter the possible changes due to the solvent effect. The NMR spectra were calculated by the Gauge Independent Atomic Orbital Approach (GIAO) in the Gaussian Program package [70,71]. Additionally, PBE0 functional was employed for the prediction of ^1^H and ^13^C NMR chemical shifts [72]. The Natural Bond Orbital Analysis [73], as implemented in the Gaussian program package, was used for the investigation of the intramolecular interactions that govern the stability of ligands and complexes. The quantum theory of atoms in molecules (QTAIM) is a complementary approach to NBO for the quantification and analysis of intramolecular interactions. This type of analysis was performed in the AIMAll program package [74] and further discussed based on Bader’s theory of interacting atoms in molecules [32,75,76]. This theory predicts two types of interactions when electron density and Laplacian of the bond critical points (BCP) and ring critical points (RCP) are concerned. The first type includes covalent (closed shell) interactions with electron density around 0.1 au and large negative Laplacian. The second type consists of ionic bonds, van der Waals interactions, and hydrogen bonds (open shell interactions), characterized by electron density values between 0.001 and 0.04 au and positive Laplacian [77].

### 3.6. Spectofluorimetric Measurements

The binding affinity of **1** towards bovine serum albumin (BSA) was investigated by spectrofluorometry on the Cary Eclipse MY2048CG03 instrument. The scan rate was set to 600 nm min^−1^, with both slits being 5 nm. The excitation wavelength characteristic for the tryptophan residues was set to 280 nm, and the emission spectrum was recorded between 300 and 500 nm. The concentration of BSA was held constant at 5 × 10^−6^ M, while the concentration of **1** changed between 1.5 and 9.9 × 10^−6^ M. The analysis followed a double logarithmical Stern-Folmer quenching. The measurements were repeated at three temperatures (30, 33, and 37 °C) to mimic body temperature.

### 3.7. Antiradical Activity

The antiradical activity of the **1** was investigated towards 2,2-diphenyl-1-picrylhydrazyl (DPPH^•^) and hydroxyl (HO^•^) radicals. The DPPH^•^ scavenging activity was measured by UV-VIS spectroscopy (Evolution 220 Thermo Scientific spectrophotometer, Waltham, MA, USA). The concentration of radical was held constant (0.1 mM) and the concentration of **1** varied from 0.1 to 10 μM, as described previously [78]. EC_50_ value was estimated as the required concentration of **1** for the reduction of 50% of the present radical. The Electron Paramagnetic Resonance (EPR) spectroscopy measurements for the anti-OH^•^ activity were performed on the Bruker Elexsys E540 EPR spectrometer (Bruker, Billerica, MA, USA) operating at X-band (9.51 GHz). The following settings were used: modulation amplitude—1G; modulation frequency—100 kHz; microwave power—100 mW. The spectra were recorded using the EXepr software (Bruker BioSpin). The samples were drawn into 10 cm long gas-permeable Teflon tubes (Zeus industries, Raritan, Franklin Township, NJ, USA) with a wall thickness of 0.025 mm and an internal diameter of 0.6 mm. The measurements were performed under normal conditions, using quartz capillaries into which Teflon tubes were placed. The Fenton system was used for the generation of HO^•^, with the following concentrations: 5 mM H_2_O_2_, 5 mM FeSO_4,_ and 100 mM spin-trap DEPMPO. The amount of radical was determined by the EPR signal after the formation of spin-adduct with DEPMPO. Due to the compound’s insolubility in water, a 15 mM solution of **1** was prepared in DMSO and diluted with water to 10 μM final concentration. The blank probe contained only the Fenton system. The radical scavenging activity of **1** was determined from the peak heights as the relative decrease of the EPR signal of spin-adduct before and after the addition of compound **1**. The activity was calculated as the % of reduction = 100 × (I_0_ − I_a_)/I_0_. In the previous equation, I_0_ and I_a_ represent the average intensities of the second and third low-field EPR peaks of the control system and a sample containing **1**, respectively.

### 3.8. Cell Culture Conditions and Viability Assays (MTT and CV)

Colon HT-29 and prostate PC-3 cell lines were obtained as a kind gift from Prof. B. Seliger (Martin Luther University Halle-Wittenberg). For maintaining the cells and the viability experiments complete medium (RPMI 1640) supplemented with FCS (10%), l-glutamine (1%), and penicillin/streptomycin (1%) was used as described somewhere else [79]. For MTT and CV experiments, HT-29, as well as PC-3, were seeded in 96 well plates at a density of 5000 cells/well and incubated for 24 h at 37 °C and 5% CO_2_ before treatment. Stock solution (20 mM) of the tested compound was diluted in 7 different working concentrations (0, 1.56, 3.12, 6.25, 12.5, 25, 50, and 100 µM). MTT and CV (Sigma Aldrich, USA) viability assays were performed as performed earlier [80]. A plate reader (Spectramax, Molecular Devices, San Jose, CA, USA) was used for absorption measurements at 570 and 670 nm [38,39]. The cell viability is represented as a percentage compared to untreated cells and the mean is calculated using a four-parametric logistic function.

### 3.9. Molecular Docking

The binding affinity of **1** towards the Bovine Serum Albumin (BSA) receptor was estimated using molecular docking simulations in the AutoDock 4.2 software [81]. The pockets and binding sites of BSA were determined by the AutoGridFR (AGFR) program. The crystal structure of BSA (PDB ID: 4OR0 [48]) was extracted from RCSB Protein Data Bank in PDB format. The target receptors were prepared for docking by removing the co-crystallized ligand, water molecules, and cofactors. For this purpose, Discovery Studio 4.0 [82] was employed. The AutoDockTools (ADT) [83] graphical user interface was used to calculate the Kollman partial charges and add the polar hydrogens. The flexibility of the ligands was analyzed, while the protein was kept as the rigid structure in the ADT. The bonds of ligands were set to be rotatable to express their flexibility. The Lamarckian Genetic Algorithm (LGA) method was applied for protein-ligand flexible docking. The parameters for the LGA method were determined as follows: the maximum number of energy evaluations was 250,000, the maximum number of generations was 27,000, and mutation and crossover rates were 0.02 and 0.8, respectively. The algorithms in the AutoDock 4.2 software were set up to predict the position of compounds within the protein target and to assess them by scoring functions by setting the grid box. The grid center with dimensions 34.75 × 24.13 × 96.47 Å^3^ in -x, -y, and -z directions of the BSA receptor was used to cover the protein binding site and accommodate ligand to move freely. For Auto Grid runs, a grid point spacing of 0.375 Å was used. The interactions between the target protein and investigated compounds as the three-dimensional (3D) results were analyzed and illustrated in Discovery Studio 4.0 and AutoDockTools.

The AutoDock program calculates these values according to the following equation, Equation (1):

(1) $$\Delta G_{bind}=\Delta G_{vdw+hbond+desolv}+\Delta G_{elec}+\Delta G_{total}+\Delta G_{tor}-\Delta G_{unb}$$

where Δ*G_bind_* is the estimated free energy of binding, the Δ*G_vdw+hbond+desolv_* denotes the sum of the energies of dispersion and repulsion (Δ*G_vdw_*), hydrogen bond (Δ*G_hbond_*), and desolvation (Δ*G_desolv_*). The Δ*G_total_* represents the final total internal energy, the Δ*G_tor_* is torsional free energy, Δ*G_unb_* is the unbound system’s energy, and Δ*G_elec_* is electrostatic energy. Ligand efficiency (LE) denotes the binding energy of ligand to protein per atom. LE (Equation (2)) has a unit of kJ mol^−1^/heavy atom.

(2) $$LE=\frac{\Delta G_{bind}}{N}$$

where *N* is the number of non-hydrogen atoms.

## 4. Conclusions

In this contribution, two new binuclear Ru(II)-arene complexes, [{RuCl(η^6^-*p*-cymene)}_2_(μ-Cl)(μ-1-*N*,*N′*-naphthyl)]X (for X = Cl (**1**) or PF_6_ (**2**)), were obtained in methanol or 2-propanol and characterized. The crystal structure of **2·2MeOH** consisted of two Ru atoms bridged by the chlorine ligand hydrazyl group. The H∙∙∙H contacts were the most important for the stabilization of crystal structure with a relative contribution of 59.7%. Other interactions, such as weak hydrogen bonds between hydrogen and fluoride/chloride, also contributed significantly. The correlation coefficients and mean absolute errors proved that the optimized structure of the cationic part of **2**, [{RuCl(η^6^-*p*-cymene)}_2_(μ-Cl)(μ-1-*N*,*N′*-naphthyl)]^+^ (**2a**) that is common for both complexes, reproduced well the crystallographic one. Various stabilization interactions govern ligand structures, as determined by the Natural Bond Orbital analysis. The interactions between donor atoms and Ru were between 240 (Ru-Cl) and 370 kJ mol^−1^ (Ru-N). Strong interaction between the *p*-cymene aromatic ring and Ru was also observed. Complementary data were obtained by the Quantum Theory of Atoms in Molecules analysis. High correlation and low MAE values were calculated when experimental and theoretical IR and NMR spectra were compared. The change in enthalpy and entropy of binding between **1** and bovine serum albumin was determined by spectrofluorimetry. These values were 189 kJ mol^−1^ and 719 J mol^−1^ K^−1^, respectively, proving that the binding process is entropically driven. The change in Gibbs free energy of binding was -34 kJ mol^−1^ at normal body temperature. These results were proven by molecular docking, with the Δ**G**_bind_ equal to -36.8 kJ mol^−1^. The most prominent interactions were hydrogen bonds, π-anion, π-cation, and π-alkyl interactions between amino acids and outer groups of **1**. The binding activity of **2a** was higher than that of naproxen, a native-bound ligand. The EC50 value of **1** towards DPPH^•^ was 0.03 mM which was comparable to kaempferol, quercetin, ascorbic acid, Trolox, and (+)-α-tocopherol. The scavenging activities of 0.5 μM solutions of **1** and ascorbic acid towards HO^•^ were 86.2 and 85.2%, respectively, again showing the high antioxidant potential of the title compound. MTT and CV assays determined the IC_50_ value of **1** against prostate PC-3 and colon HT-29 cancer cell lines (IC_50_ [µM] on PC-3 40.52 ± 3.89 (MTT), 16.92 ± 1.42 (CV); HT-29 37.90 ± 2.33 (MTT), 11.49 ± 1.87 (CV)). The inhibition concentration was lower than the respective concentration for cisplatin, although the higher tolerance of ruthenium in biological systems might indicate potential applicability. The high stability, binding affinity towards BSA, antiradical, and cytotoxic activities of **1** make it a promising candidate for future biochemical studies and possible applications.

## Figures and Tables

**Table 1:** Crystal data and structure refinement details of **2·2MeOH**.

Empirical formula	C_32_H_46_Cl_3_F_6_N_2_O_2_PRu
Formula weight [g mol^−1^]	944.17
Temperature [K]	100
Crystal System	monoclinic
Space group	*I_2_/a*
Radiation/Wavelength [Å]	Mo K_a_/0.71073
Unit cell dimension [Å, deg]	a = 21.3209(5), α = 90° b = 10.5848(2), β = 99.702(2)° c = 33.3109(8), γ = 90
Volume	7410.0(3) Å^3^
Calculated density	1.691 Mg m^−3^
Z	8
Θ range [deg]	3.116–29.015
Reflections	
Measured:	41,874
Independent:	8907
Observed [I > 2s(I)]:	7696
*R_int_*	0.0411
*R_1_ [I > 2s(I)], R_1_ (all)*	0.0354, 0.0442
*wR_2_ [I > 2s(I)], wR_2_ (all)*	0.0827, 0.0883
Goodness-of-fit on *F^2^*	1.037
Largest diff. peak/hole (e Å^−3^)	1.032/−0.720
CCDC no.	2217194

**Table 2:** Selected bond lengths (A) and angles (°) of the hydrogen bridges formed by 2**·2MeOH** in the solid state.

D−H^...^Acceptor	D^...^A	D−H^...^A
*Intramolecular Type*		
N2−H3N^...^Cl1	3.073	138
N1−H1N^...^Cl3	3.144	126
*Intermolecular Type*		
N1−H1N^...^F5	3.322	167
O2M−H2M^...^O1M	2.801	156
N1−H2N^...^O2M	2.826	156

Symmetry code: “A” = x, y + 1, z.

**Table 3:** Thermodynamic parameters of binding of **1** to BSA at different temperatures.

T [K]	K_b_ [M^−1^]	ΔH_b_ [kJ mol^−1^]	ΔS_b_ [J mol^−1^ K^−1^]	ΔG_b_ [kJ mol^−1^]	R
303	9.90 × 10^4^	189	719	−29	0.998
306	2.03 × 10^5^			−31	
310	5.37 × 10^5^			−34	

**Table 4:** The important thermodynamic parameters for best docking conformations of investigated molecules with BSA (PDB ID: 4OR0).

	ΔG_bind_ (kJ mol^−1^)	ΔG_vdw+hbond+desolv_ (kJ mol^−1^)	ΔG_elec_ (kJ mol^−1^)	ΔG_total_ (kJ mol^−1^)	ΔG_tor_ (kJ mol^−1^)	ΔG_unb_ (kJ mol^−1^)	LE
**NPS**-BSA	−21.3	−24.5	0.3	−1.5	2.8	−1.5	−0.3
**1**-BSA	−36.8	−26.3	−15.3	−7.7	4.8	−7.7	−0.2

## Data Availability

Not applicable.

## Supplementary Materials

The following supporting information can be downloaded at: https://www.mdpi.com/article/10.3390/ijms24010689/s1.

## References

[1] Kenny R.G., Marmion C.J. (2019). Toward Multi-Targeted Platinum and Ruthenium Drugs—A New Paradigm in Cancer Drug Treatment Regimens?. Chem. Rev..

[2] Johnstone T.C., Suntharalingam K., Lippard S.J. (2016). The Next Generation of Platinum Drugs: Targeted Pt(II) Agents, Nanoparticle Delivery, and Pt(IV) Prodrugs. Chem. Rev..

[3] Thota S., Rodrigues D.A., Crans D.C., Barreiro E.J. (2018). Ru(II) Compounds: Next-Generation Anticancer Metallotherapeutics?. J. Med. Chem..

[4] Simović A.R., Masnikosa R., Bratsos I., Alessio E. (2019). Chemistry and reactivity of ruthenium(II) complexes: DNA/protein binding mode and anticancer activity are related to the complex structure. Coord. Chem. Rev..

[5] Conti L., Macedi E., Giorgi C., Valtancoli B., Fusi V. (2022). Combination of light and Ru(II) polypyridyl complexes: Recent advances in the development of new anticancer drugs. Coord. Chem. Rev..

[6] Lee S.Y., Kim C.Y., Nam T.-G. (2020). Ruthenium Complexes as Anticancer Agents: A Brief History and Perspectives. Drug Des. Dev. Ther..

[7] Durig J.R., Danneman J., Behnke W.D., Mercer E.E. (1976). The induction of filamentous growth in *Escherichia coli* by ruthenium and palladium complexes. Chem. Biol. Interact..

[8] Therrien B. (2009). Functionalised η^6^-arene ruthenium complexes. Coord. Chem. Rev..

[9] Clarke M.J. (2002). Ruthenium metallopharmaceuticals. Coord. Chem. Rev..

[10] Smith G.S., Therrien B. (2011). Targeted and multifunctional arene ruthenium chemotherapeutics. Dalton Trans..

[11] Kawano M., Hoshino C., Matsumoto K. (1992). Synthesis, properties, and crystal structure of a novel μ-hydrazine-bridged mixed-valence ruthenium(II,III) complex stabilized by hydrazine hydrogen bonds, [RuCl(TMP)_2_]_2_(μ-Cl)(μ-N_2_H_4_)(μ-S_2_) (TMP=trimethylphosphite). Inorg. Chem..

[12] Rozenel S.S., Arnold J. (2012). Bimetallic Ruthenium PNP Pincer Complex as a Platform to Model Proposed Intermediates in Dinitrogen Reduction to Ammonia. Inorg. Chem..

[13] Matsumoto K., Koyama T., Koide Y. (1999). Oxidation of the Sulfide Ligands to SO_4_^2−^ in the Dinuclear Complex {RuCl[P(OMe)_3_]_2_}_2_(μ-S_2_)(μ-Cl)(μ-N_2_H_4_): Synthesis and Characterization of {RuCl[P(OMe)_3_]_2_}_2_(μ-S_2_)(μ-Cl)(μ-N_2_H_4_)^+^HSO_4_^−^, {RuCl_2_[P(OMe)_3_]_2_}_2_(μ-S)(μ-N_2_H_4_), and {RuCl_2_[P(OMe)_3_]_2_}_2_(μ-S_2_O_5_)(μ-N_2_H_4_). J. Am. Chem. Soc..

[14] Mashima K., Kaneko S., Tani K., Kaneyoshi H., Nakamura A. (1997). Synthesis and reactions of coordinatively unsaturated 16-electron chalcogenolate complexes, Ru(EAr)_2_(η^6^-arene) and cationic binuclear chalcogenolate complexes, [(η^6^-arene) Ru(μ-EPh)_3_Ru(η^6^-arene)]PF_6_. J. Organomet. Chem..

[15] Ashworth T.V., Nolte M.J., Reimann R.H., Singleton E. (1977). Dimeric ruthenium(II) complexes with bridging NN-dimethylhydrazine ligands: X-ray analysis of the structure of [{RuHCl(cyclo-octa-1,5-diene)}_2_NH_2_NMe_2_]. J. Chem. Soc. Chem. Commun..

[16] Ashworth T.V., Singleton E., Hough J.J. (1977). Cationic ruthenium(II) systems. Part 1. The preparation and reactivity of diene(hydrazine)ruthenium(II) cations, and the formation of aminobonded hydrazone complexes. J. Chem. Soc. Dalton Trans..

[17] Albertin G., Antoniutti S., Botter A., Castro J. (2014). Hydrazine complexes of ruthenium with cyclopentadienyl and indenyl ligands: Preparation and reactivity. J. Organomet. Chem..

[18] Reindl S.A., Pöthig A., Drees M., Bechlars B., Herdtweck E., Herrmann W.A., Kühn F.E. (2013). Pyrazolato-Bridged Dinuclear Complexes of Ruthenium(II) and Rhodium(III) with N-Heterocyclic Carbene Ligands: Synthesis, Characterization, and Electrochemical Properties. Organometallics.

[19] Rigby W., McCleverty J.A., Maitlis P.M. (1979). Pentamethylcyclopentadienyl-rhodium and -iridium complexes. Part 20. Rhodium complexes of co-ordinated hydrazines. J. Chem. Soc. Dalton Trans..

[20] Pal S., Pal S. (2001). A Diruthenum(III) Complex Possessing a Diazine and Two Chloride Bridges: Synthesis, Structure, and Properties. Inorg. Chem..

[21] Matsumoto K., Uemura H., Kawano M. (1994). A Novel Diruthenium(II,III) Complex, [{Ru(AN)(TMP)_2_}_2_(μ-S_2_)(μ-NH_2_NH_2_)_2_](CF_3_SO_3_)_3_ ·Et_2_O, with Two Hydrazine Bridges (AN=acetonitrile, TMP=P(OMe)_3_). Chem. Lett..

[22] Lalrempuia R., Kollipara M.R., Carroll P.J. (2003). Syntheses and characterization of arene ruthenium (II) complexes containing N,N′-donor Schiff base ligands. Crystal and molecular structure of [(η^6^-C_6_Me_6_)Ru(C_5_H_4_N–2–CH=N–C_6_H_4_–p–NO_2_)]PF. Polyhedron.

[23] Al-Noaimi M., AlDamen M.A. (2012). Ruthenium complexes incorporating azoimine and α-diamine based ligands: Synthesis, crystal structure, electrochemistry and DFT calculation. Inorg. Chim. Acta.

[24] Uahengo V., Cai P., Naimhwaka J., Rahman A., Daniel L.S., Bhakhoa H., Rhyman L., Ramasami P. (2019). Photophysical, electrochemical, and DFT studies of the novel azacrown-bridged dinuclear ruthenium dye sensitizers for solar cells. Polyhedron.

[25] Sikalov A.A., Pavlovskiy V.V., Kirilchuk A.A. (2019). Ruthenium(II) p-cymene complexes bearing 2-(1,2,4-triazol-3-yl)pyridines: Linkage isomerism and related NMR/DFT studies. Inorg. Chem. Commun..

[26] Mondal A., Sen U., Roy N., Muthukumar V., Sahoo S.K., Bose B., Paira P. (2021). DNA targeting half sandwich Ru(II)-*p*-cymene-N^N complexes as cancer cell imaging and terminating agents: Influence of regioisomers in cytotoxicity. Dalton Trans..

[27] Rupp M.T., Shevchenko N., Hanan G.S., Kurth D.G. (2021). Enhancing the photophysical properties of Ru(II) complexes by specific design of tridentate ligands. Coord. Chem. Rev..

[28] Cabeza J.A., Van der Maelen J.F., García-Granda S. (2009). Topological Analysis of the Electron Density in the N-Heterocyclic Carbene Triruthenium Cluster [Ru_3_(μ-H)_2_(μ_3_-MeImCH)(CO)_9_](Me_2_Im=1,3-dimethylimidazol-2-ylidene). Organometallics.

[29] Firme C.L., de Pontes D.L., Antunes O.A.C. (2010). Topological study of bis(cyclopentadienyl) titanium and bent titanocenes. Chem. Phys. Lett..

[30] Pocha R., Löhnert C., Johrendt D. (2007). The metal-rich palladium chalcogenides Pd_2_MCh_2_ (M=Fe, Co, Ni; Ch=Se, Te): Crystal structure and topology of the electron density. J. Solid State Chem..

[31] Gholivand K., Mahzouni H.R., Esrafili M.D. (2012). Structure, bonding, electronic and energy aspects of a new family of early lanthanide (La, Ce and Nd) complexes with phosphoric triamides: Insights from experimental and DFT studies. Dalton Trans..

[32] Bader R.F.W. (1998). A Bond Path:  A Universal Indicator of Bonded Interactions. J. Phys. Chem. A.

[33] Dindar S., Nemati Kharat A., Safarkoopayeh B., Abbasi A. (2021). Ruthenium(II)β-diketimine as hydroamination catalyst, crystal structure and DFT computations. Transit. Met. Chem..

[34] Zahirović A., Roca S., Kahrović E., Višnjevac A. (2021). Low DNA and high BSA binding affinity of cationic ruthenium(II) organometallic featuring pyridine and 2′-hydroxychalcone ligands. J. Mol. Struct..

[35] Nakamoto K. (1997). Infrared and Ramon Spectra of Inorganic and Coordination Compounds.

[36] Turan N., Buldurun K. (2018). Synthesis, characterization and antioxidant activity of Schiff base and its metal complexes with Fe(II), Mn(II), Zn(II), and Ru(II) ions: Catalytic activity of ruthenium(II) complex. Eur. J. Chem..

[37] Durig J.R., McAllister W.A., Mercer E.E. (1967). On the I.R. spectra of the isomers of trichlorotriaquoruthenium(III). J. Inorg. Nucl. Chem..

[38] Arlt S., Petković V., Ludwig G., Eichhorn T., Lang H., Rüffer T., Mijatović S., Maksimović-Ivanić D., Kaluđerović G.N. (2021). Arene Ruthenium(II) Complexes Bearing the κ-P or κ-P, κ-S Ph_2_P(CH_2_)_3_SPh Ligand. Molecules.

[39] Jeremias L., Novotný J., Repisky M., Komorovsky S., Marek R. (2018). Interplay of Through-Bond Hyperfine and Substituent Effects on the NMR Chemical Shifts in Ru(III) Complexes. Inorg. Chem..

[40] Kulkarni A.D., Truhlar D.G. (2011). Performance of Density Functional Theory and Møller–Plesset Second-Order Perturbation Theory for Structural Parameters in Complexes of Ru. J. Chem. Theory Comput..

[41] Novotný J., Přichystal D., Sojka M., Komorovsky S., Nečas M., Marek R. (2018). Hyperfine Effects in Ligand NMR: Paramagnetic Ru(III) Complexes with 3-Substituted Pyridines. Inorg. Chem..

[42] Novotný J., Sojka M., Komorovsky S., Nečas M., Marek R. (2016). Interpreting the Paramagnetic NMR Spectra of Potential Ru(III) Metallodrugs: Synergy between Experiment and Relativistic DFT Calculations. J. Am. Chem. Soc..

[43] Zahirović A., Žilić D., Pavelić S.K., Hukić M., Muratović S., Harej A., Kahrović E. (2019). Type of complex–BSA binding forces affected by different coordination modes of alliin in novel water-soluble ruthenium complexes. New J. Chem..

[44] Xiao Q., Huang S., Liu Y., Tian F., Zhu J. (2009). Thermodynamics, Conformation and Active Sites of the Binding of Zn–Nd Hetero-bimetallic Schiff Base to Bovine Serum Albumin. J. Fluoresc..

[45] Devagi G., Dallemer F., Kalaivani P., Prabhakaran R. (2018). Organometallic ruthenium(II) complexes containing NS donor Schiff bases: Synthesis, structure, electrochemistry, DNA/BSA binding, DNA cleavage, radical scavenging and antibacterial activities. J. Organomet. Chem..

[46] Khan T.A., Bhar K., Thirumoorthi R., Roy T.K., Sharma A.K. (2020). Design, synthesis, characterization and evaluation of the anticancer activity of water-soluble half-sandwich ruthenium(II) arene halido complexes. New J. Chem..

[47] Kamatchi T.S., Kalaivani P., Poornima P., Padma V.V., Fronczek F.R., Natarajan K. (2014). New organometallic ruthenium( II) complexes containing chelidonic acid (4-oxo-4H-pyran-2,6-dicarboxylic acid): Synthesis, structure and in vitro biological activity. RSC Adv..

[48] Bujacz A., Zielinski K., Sekula B. (2014). Structural studies of bovine, equine, and leporine serum albumin complexes with naproxen. Proteins Struct. Funct. Bioinform..

[49] Foti M.C. (2015). Use and Abuse of the DPPH^•^ Radical. J. Agric. Food Chem..

[50] Sârbu C., Casoni D. (2013). Comprehensive evaluation of biogenic amines and related drugs’ antiradical activity using reactive 2,2-diphenyl-1-picrylhydrazyl (DPPH) radical. Open Chem..

[51] Foti M.C., Daquino C., Mackie I.D., DiLabio G.A., Ingold K.U. (2008). Reaction of Phenols with the 2,2-Diphenyl-1-picrylhydrazyl Radical. Kinetics and DFT Calculations Applied To Determine ArO-H Bond Dissociation Enthalpies and Reaction Mechanism. J. Org. Chem..

[52] Foti M.C., Daquino C., DiLabio G.A., Ingold K.U. (2011). Kinetics of the Oxidation of Quercetin by 2,2-Diphenyl-1-picrylhydrazyl (dpph**^•^**). Org. Lett..

[53] Foti M.C., Daquino C., Geraci C. (2004). Electron-Transfer Reaction of Cinnamic Acids and Their Methyl Esters with the DPPH^•^ Radical in Alcoholic Solutions. J. Org. Chem..

[54] Dimić D., Milenković D., Marković J.D., Marković Z. (2017). Antiradical activity of catecholamines and metabolites of dopamine: Theoretical and experimental study. Phys. Chem. Chem. Phys..

[55] Singh G., Mohanty B.P., Saini G.S.S. (2016). Structure, spectra and antioxidant action of ascorbic acid studied by density functional theory, Raman spectroscopic and nuclear magnetic resonance techniques. Spectrochim. Acta Part A Mol. Biomol. Spectrosc..

[56] Allen R.N., Shukla M.K., Reed D., Leszczynski J. (2006). Ab initio study of the structural properties of ascorbic acid (vitamin C). Int. J. Quantum Chem..

[57] Milenković D.A., Dimić D.S., Avdović E.H., Amić A.D., Marković J.M.D., Marković Z.S. (2020). Advanced oxidation process of coumarins by hydroxyl radical: Towards the new mechanism leading to less toxic products. Chem. Eng. J..

[58] Smolko L., Smolková R., Samoľová E., Morgan I., Saoud M., Kaluđerović G.N. (2020). Two isostructural Co(II) flufenamato and niflumato complexes with bathocuproine: Analogues with a different cytotoxic activity. J. Inorg. Biochem..

[59] (2017). CrysAlisPRO.

[60] Turner M.J., McKinnon J.J., Wolff S.K., Grimwood D.J., Spackman P.R., Jayatilaka D., Spackman M.A. (2017). CrystalExplorer17.

[61] Shobana D., Sudha S., Ramarajan D., Dimić D. (2022). Synthesis, crystal structure, spectral characterization and Hirshfeld surface analysis of (E)-N′-(3-ethoxy-4-hydroxybenzylidene)-4-fluorobenzohydrazide single-crystal—A novel NLO active material. J. Mol. Struct..

[62] Spackman M.A., Byrom P.G. (1997). A novel definition of a molecule in a crystal. Chem. Phys. Lett..

[63] Spackman M.A., Jayatilaka D. (2009). Hirshfeld surface analysis. CrystEngComm.

[64] Frisch M.J., Trucks G.W., Schlegel H.B., Scuseria G.E., Robb M.A., Cheeseman J.R., Scalmani G., Barone V., Mennucci B., Petersson G.A. (2009). Gaussian 09, Revision C.01.

[65] Becke A.D. (1993). Density-functional thermochemistry. III. The role of exact exchange. J. Chem. Phys..

[66] Dunning T.H. (1989). Gaussian basis sets for use in correlated molecular calculations. I. The atoms boron through neon and hydrogen. J. Chem. Phys..

[67] Hay P.J., Wadt W.R. (1985). Ab initio effective core potentials for molecular calculations. Potentials for the transition metal atoms Sc to Hg. J. Chem. Phys..

[68] Hay P.J., Wadt W.R. (1985). Ab initio effective core potentials for molecular calculations. Potentials for K to Au including the outermost core orbitale. J. Chem. Phys..

[69] Marenich A.V., Cramer C.J., Truhlar D.G. (2009). Universal Solvation Model Based on Solute Electron Density and on a Continuum Model of the Solvent Defined by the Bulk Dielectric Constant and Atomic Surface Tensions. J. Phys. Chem. B.

[70] Zieliński R., Szymusiak H. (2003). Application of Dft B3Lyp/Giao and B3Lyp/Csgt Methods for Interpretation of Nmr Spectra of Flavonoids. Pol. J. Food Nutr. Sci..

[71] Bohmann J.A., Weinhold F., Farrar T.C. (1997). Natural chemical shielding analysis of nuclear magnetic resonance shielding tensors from gauge-including atomic orbital calculations. J. Chem. Phys..

[72] Adamo C., Barone V. (1999). Toward reliable density functional methods without adjustable parameters: The PBE0 model. J. Chem. Phys..

[73] Reed A.E., Weinstock R.B., Weinhold F. (1985). Natural population analysis. J. Chem. Phys..

[74] Keith T.A. (2019). TK Gristmill Software.

[75] Bader R.F.W. (1985). Atoms in molecules. Acc. Chem. Res..

[76] Bader R.F.W. (1990). Atoms in Molecules: A Quantum Theory.

[77] Dimić D., Petković M. (2016). Control of a photoswitching chelator by metal ions: DFT, NBO, and QTAIM analysis. Int. J. Quantum Chem..

[78] Nenadis N., Tsimidou M. (2002). Observations on the estimation of scavenging activity of phenolic compounds using rapid 1, 1-diphenyl-2-picrylhydrazyl (DPPH•) tests. J. Am. Oil Chem. Soc..

[79] Krajnović T., Pantelić N.Đ., Wolf K., Eichhorn T., Maksimović-Ivanić D., Mijatović S., Wessjohann L.A., Kaluđerović G.N. (2022). Anticancer Potential of Xanthohumol and Isoxanthohumol Loaded into SBA-15 Mesoporous Silica Particles against B16F10 Melanoma Cells. Materials.

[80] Morgan I., Wessjohann L.A., Kaluđerović G.N. (2022). In Vitro Anticancer Screening and Preliminary Mechanistic Study of A-Ring Substituted Anthraquinone Derivatives. Cells.

[81] Morris G.M., Huey R., Lindstrom W., Sanner M.F., Belew R.K., Goodsell D.S., Olson A.J. (2009). AutoDock4 and AutoDockTools4: Automated docking with selective receptor flexibility. J. Comput. Chem..

[82] BIOVIA (2016). Dassault Systèmes, Discovery Studio 2016.

[83] Rosselli S., Maggio A.M., Faraone N., Spadaro V., Morris-Natschke S.L., Bastow K.F., Lee K.-H., Bruno M. (2009). The cytotoxic properties of natural coumarins isolated from roots of Ferulago campestris (Apiaceae) and of synthetic ester derivatives of aegelinol. Nat. Prod. Commun..

