# Supplementary material for: Synthesis, Crystallographic Structure, Theoretical Analysis, Molecular Docking Studies, and Biological Activity Evaluation of Binuclear Ru(II)-1-Naphthylhydrazine Complex
Source: Int J Mol Sci. 2022 Dec 30;24(1):689. doi: 10.3390/ijms24010689 (PMC9821167; doi:10.3390/ijms24010689)
Supplement: Supplementary file 1 [file ijms-24-00689-s001.zip › ijms-2069915-supplementary.pdf]

Supplementary Information for:

Synthesis, Crystallographic Structure, Theoretical Analysis, Molecular Docking Studies, and Biological Activity Evaluation of Binuclear Ru(II)-1-Naphthylhydrazine Complex

Thomas Eichhorn [1], Franz Kolbe [1], Stefan Mišić [1,2], Dušan Dimić [2], Ibrahim Morgan [3], Mohamad Saoud [3], Dejan Milenković [4], Zoran Marković [4], Tobias Rüffer [5], Jasmina Dimitrić Marković [2,*] and Goran N. Kaluđerović [1,*]

[1] Department of Engineering and Natural Sciences, University of Applied Sciences Merseburg, Eberhard-Leibnitz-Straße 2, 06217 Merseburg, Germany
[2] Faculty of Physical Chemistry, University of Belgrade, Studentski trg 12-16, 11000 Belgrade, Serbia
[3] Department of Bioorganic Chemistry, Leibniz Institute of Plant Biochemistry, Weinberg 3, 06120 Halle (Saale), Germany
[4] Department of Science, Institute for Information Technologies, University of Kragujevac, Jovana Cvijića bb, 34000 Kragujevac, Serbia
[5] Institute of Chemistry, Chemnitz University of Technology, Straße der Nationen 62, D-09111 Chemnitz, Germany
* Correspondence: markovich@ffh.bg.ac.rs (J.D.M.); goran.kaluderovic@hs-merseburg.de (G.N.K.)

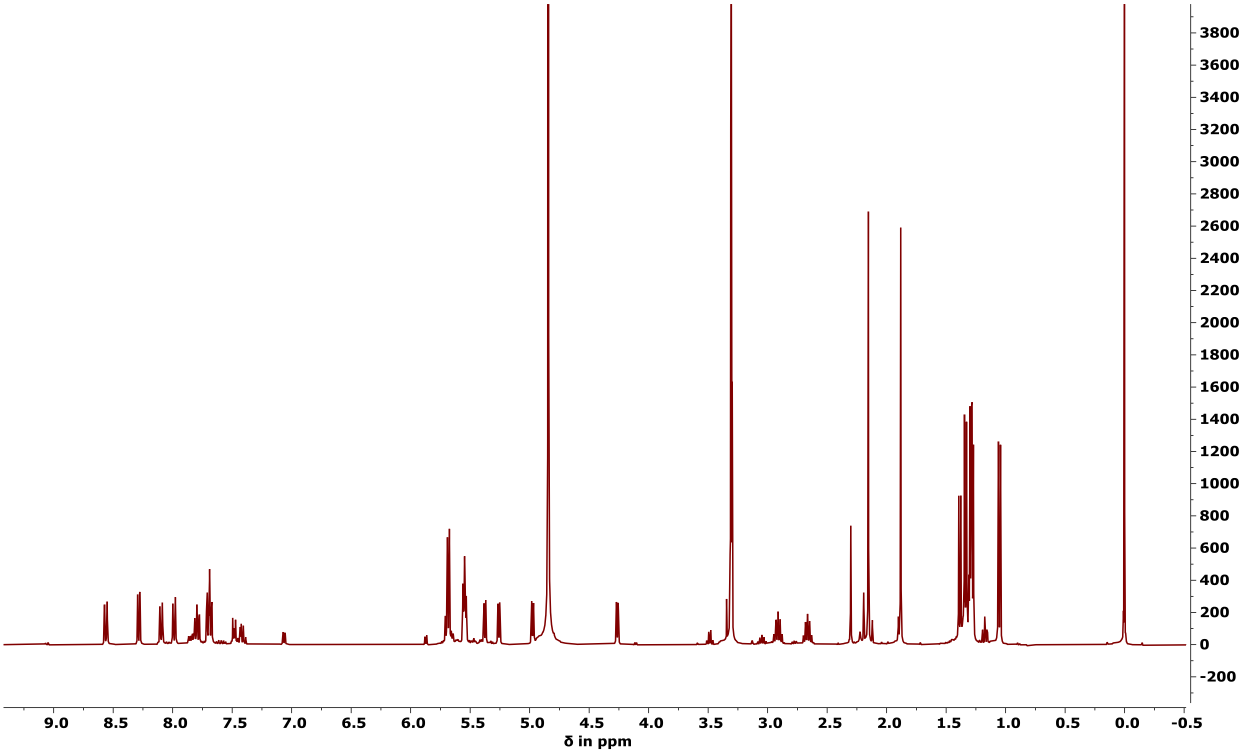

Figure S1. ^{1}H NMR spectrum of **1**

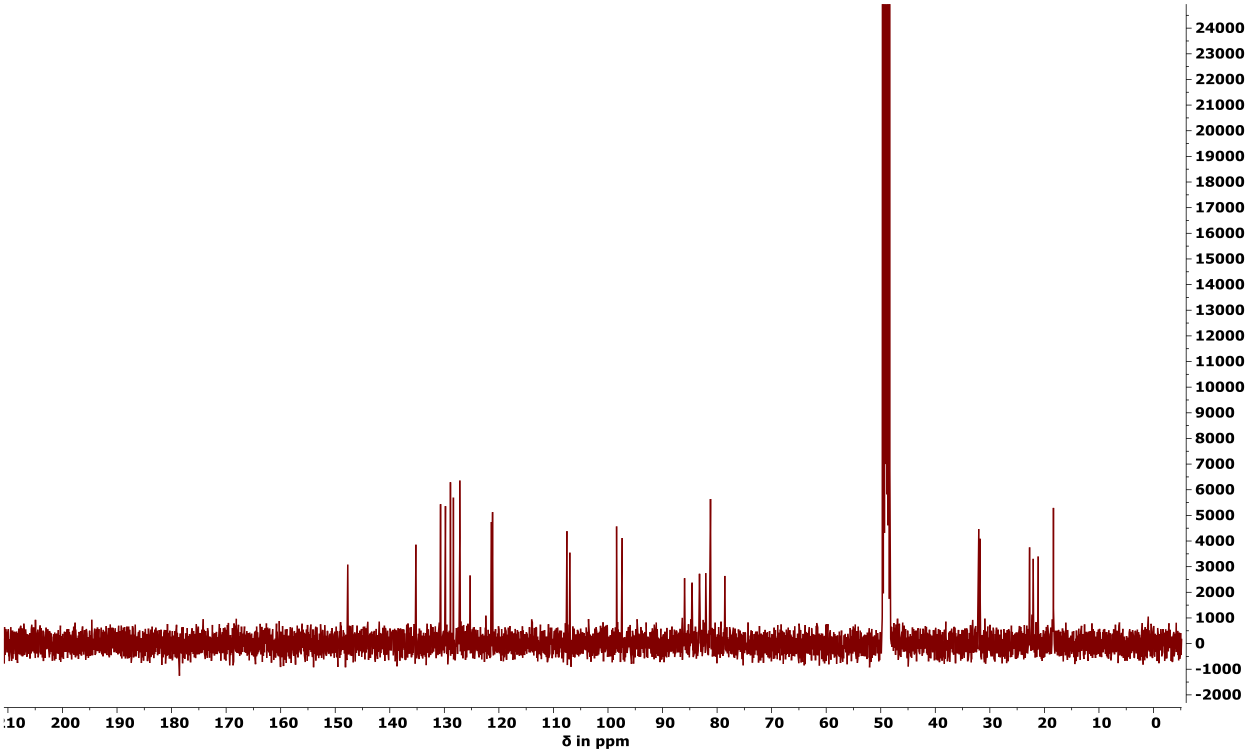

Figure S2. ^{13}C NMR spectrum of **1**

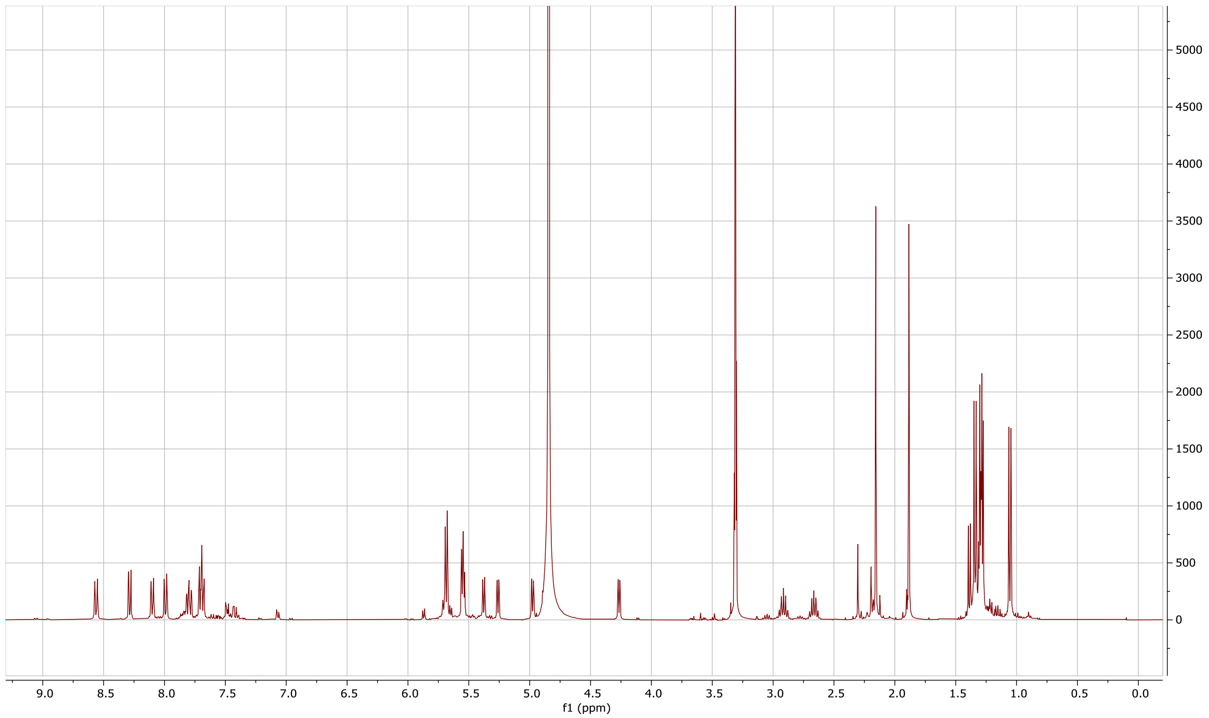

Figure S3. ^{1}H NMR spectrum of **2**

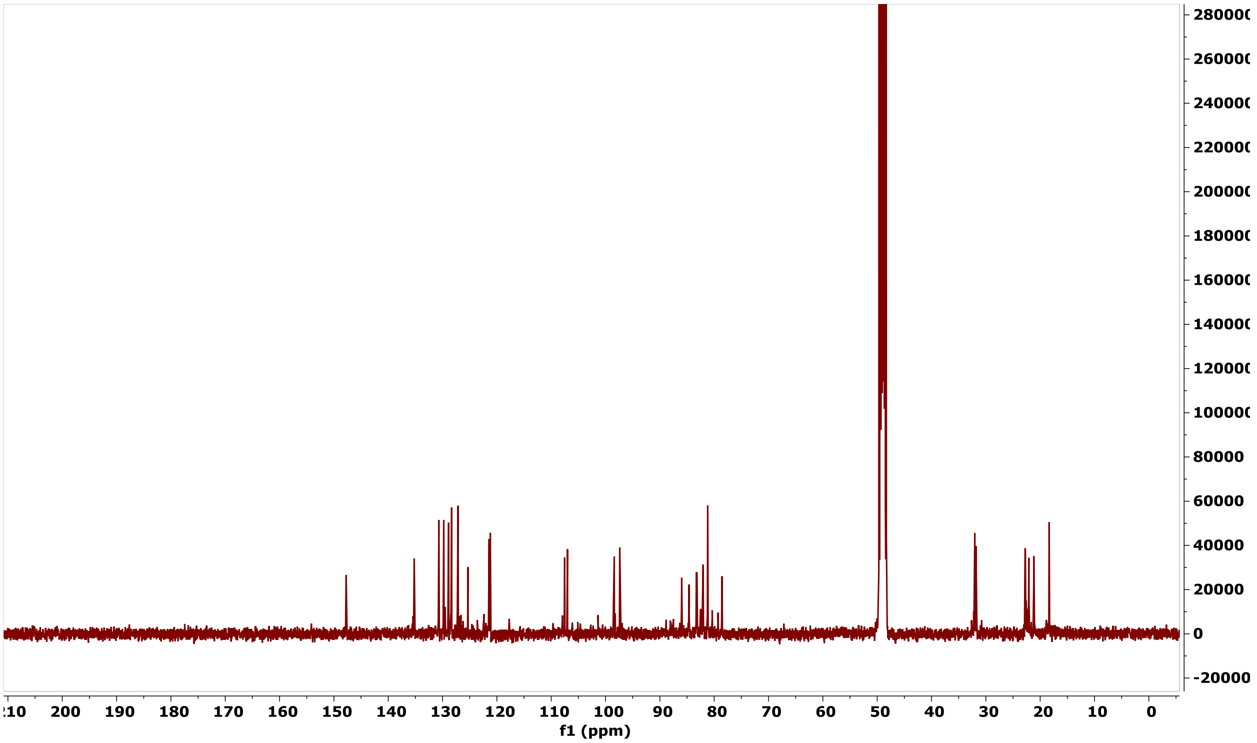

Figure S4. ^{13}C NMR spectrum of **2**

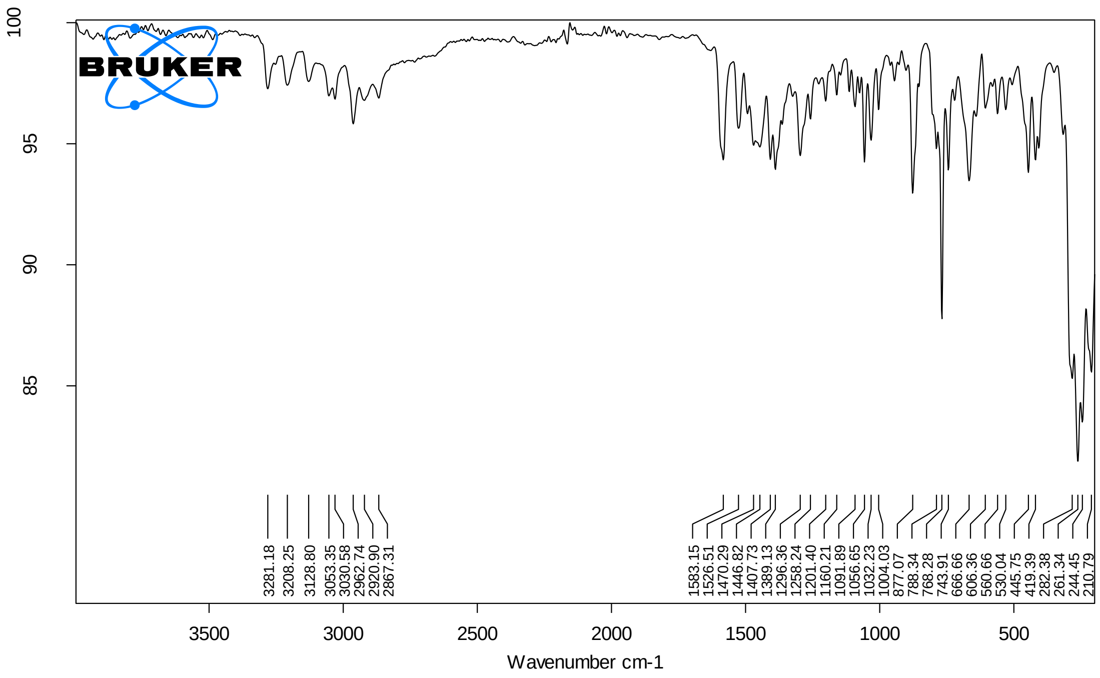

Figure S5. IR spectrum of **2**

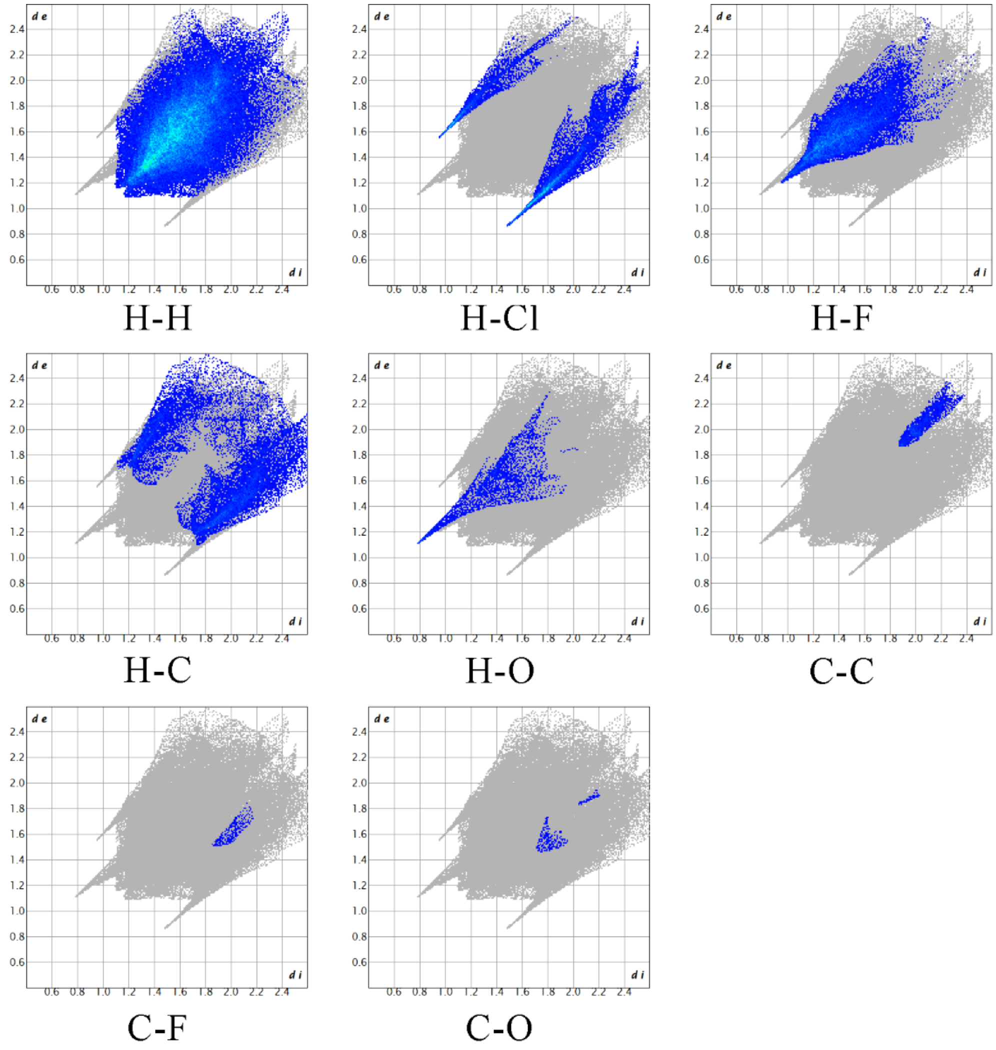

Figure S6. The fingerprint plots for the most abundant contacts in crystal structure of **2·2MeOH**.

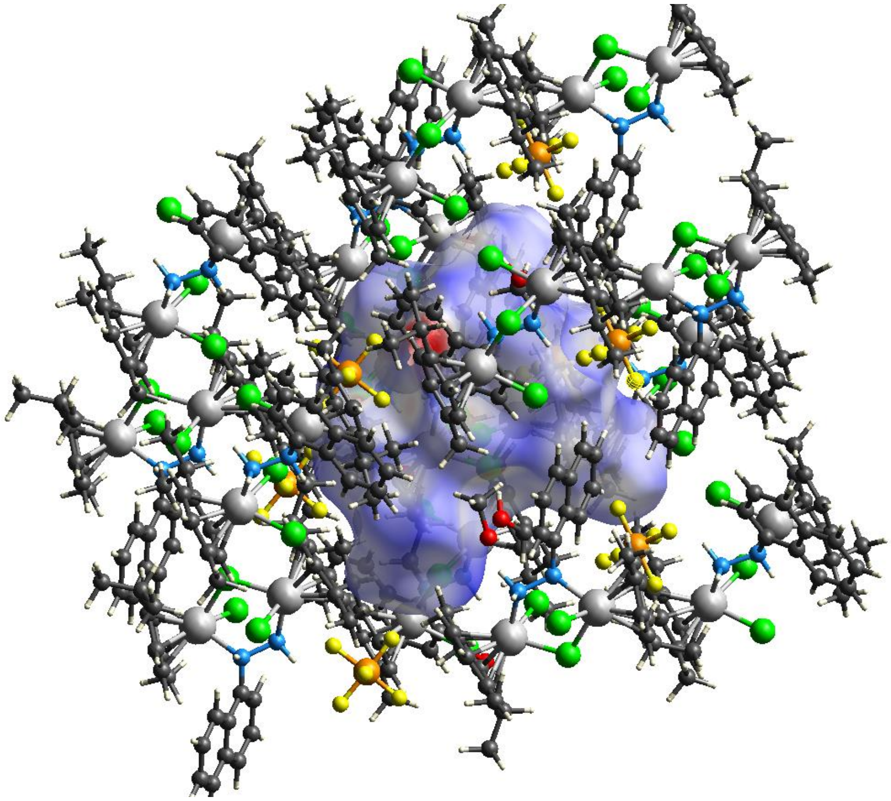

Figure S7. Hirshfeld surface of **2a** with surrounding units.

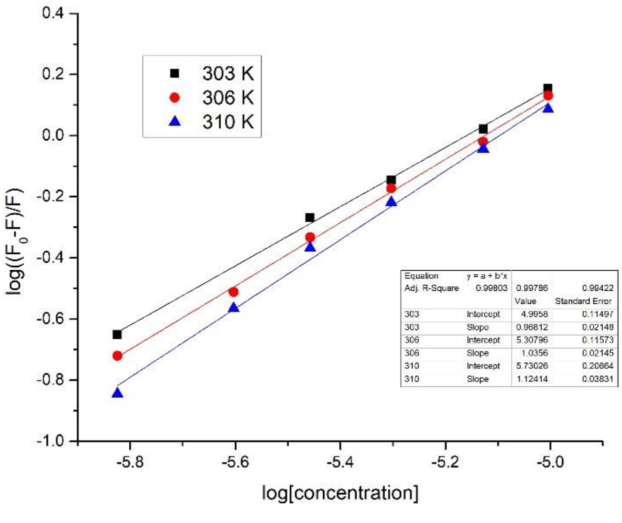

Figure S8. Stern-Volmer plots for the binding of **1** to BSA.

Table S1. The experimental and theoretical bond lengths of **2a** (in Å), the notations follow Figure 1.

Bond	Experimental	Theoretical
C1−C2	1.402(5)	1.440
C1−C6	1.434(5)	1.414
C1−C7	1.508(5)	1.502
C2−C3	1.425(4)	1.406
C3−C4	1.404(4)	1.443
C4−C5	1.435(4)	1.410
C4−C8	1.523(4)	1.517
C5−C6	1.392(5)	1.436
C8−C9	1.513(5)	1.533
C9−C10	1.555(5)	1.547
C11−C16	1.404(5)	1.413
C11−C12	1.430(5)	1.440
C11−C17	1.508(5)	1.501
C12−C13	1.401(5)	1.406
C13−C14	1.431(4)	1.443
C14−C15	1.413(4)	1.411
C14−C18	1.516(4)	1.517
C15−C16	1.422(4)	1.411
C18−C20	1.519(4)	1.533
C18−C19	1.544(4)	1.546
C21−C22	1.371(4)	1.382
C21−C30	1.425(4)	1.433
C21−N2	1.465(3)	1.460
C22−C23	1.410(4)	1.412
C23−C24	1.369(4)	1.375
C24−C25	1.422(4)	1.420
C25−C26	1.411(4)	1.421
C25−C30	1.435(4)	1.439
C26−C27	1.366(4)	1.375
C27−C28	1.416(4)	1.414
C28−C29	1.373(4)	1.379
C29−C30	1.423(4)	1.425
N1−N2	1.467(3)	1.465
N1−Ru1	2.138(2)	2.189
N2−Ru2	2.193(2)	2.238
Cl1−Ru1	2.4063(7)	2.444
Cl2−Ru2	2.4172(7)	2.482
Cl2−Ru1	2.4287(7)	2.487

Cl3−Ru2	2.4150(7)	2.462
C1−Ru1	2.196(3)	2.274
C2−Ru1	2.161(3)	2.229
C3−Ru1	2.178(3)	2.241
C4−Ru1	2.216(3)	2.321
C5−Ru1	2.171(3)	2.279
C6−Ru1	2.171(3)	2.239
C11−Ru2	2.203(3)	2.276
C12−Ru2	2.165(3)	2.247
C13−Ru2	2.184(3)	2.275
C14−Ru2	2.226(3)	2.311
C15−Ru	2.198(3)	2.238
C16−Ru	2.175(3)	2.230
R		0.999
MAE [Å]		0.031

Table S2. The experimental and theoretical bond angles of **2a** (in ^{o}), the notations follow Figure 1.

Angle	Experimental	Theoretical
C2−C1−C6	117.2(3)	116.9
C2−C1−C7	121.7(3)	120.6
C6−C1−C7	121.1(3)	122.4
C2−C1−Ru1	69.91(17)	69.7
C6−C1−Ru	69.89(17)	70.4
C7−C1−Ru1	128.5(2)	128.7
C1−C2−C3	121.7(3)	121.2
C1−C2−Ru1	72.57(18)	73.07
C3−C2−Ru1	71.47(16)	72.11
C4−C3−C2	121.2(3)	121.7
C4−C3−Ru1	72.82(16)	74.60
C2−C3−Ru1	70.18(16)	71.21
C3−C4−C5	116.9(3)	117.1
C3−C4−C8	123.1(3)	119.1
C5−C4−C8	120.0(3)	123.8
C3−C4−Ru1	69.93(15)	68.57
C5−C4−Ru1	69.22(15)	70.53
C8−C4−Ru1	129.3(2)	131.5
C6−C5−C4	122.0(3)	121.0

C6−C5−Ru1	71.29(16)	69.99
C4−C5−Ru1	72.60(15)	73.77
C5−C6−C1	121.0(3)	121.9
C5−C6−Ru1	71.31(16)	72.96
C1−C6−Ru1	71.78(16)	73.08
C9−C8−C4	113.7(3)	114.5
C9−C8−C10	110.0(3)	111.2
C4−C8−C10	108.5(3)	108.6
C16−C11−C12	116.8(3)	117.0
C16−C11−C17	121.7(3)	120.8
C12−C11−C17	121.5(3)	122.1
C16−C11−Ru2	70.19(16)	70.67
C12−C11−Ru2	69.45(17)	69.61
C17−C11−Ru2	129.3(2)	128.8
C13−C12−C11	121.6(3)	121.1
C13−C12−Ru2	71.93(17)	71.98
C11−C12−Ru2	72.35(17)	73.13
C12−C13−C14	121.0(3)	121.8
C12−C13−Ru2	70.49(17)	71.33
C14−C13−Ru2	72.70(16)	74.29
C15−C14−C13	117.9(3)	117.0
C15−C14−C18	123.3(3)	123.9
C13−C14−C18	118.8(3)	119.1
C15−C14−Ru2	70.29(16)	70.66
C13−C14−Ru2	69.46(16)	68.78
C18−C14−Ru2	131.3(2)	131.3
C14−C15−C16	120.2(3)	121.2
C14−C15−Ru2	72.47(16)	73.51
C16−C15−Ru2	70.13(16)	70.46
C11−C16−C15	122.4(3)	121.8
C11−C16−Ru2	72.41(17)	72.94
C15−C16−Ru2	71.93(16)	72.55
C14−C18−C20	113.9(3)	114.5
C14−C18−C19	107.3(3)	108.6
C20−C18−C19	111.4(3)	111.3
C22−C21−C30	121.9(2)	121.3
C22−C21−N2	119.0(2)	119.0
C30−C21−N2	119.1(2)	119.6
C21−C22−C23	120.1(3)	120.4
C24−C23−C22	120.5(3)	120.2
C23−C24−C25	120.6(3)	120.8

C26−C25−C24	121.3(2)	120.9
C26−C25−C30	119.0(3)	119.2
C24−C25−C30	119.7(2)	119.9
C27−C26−C25	121.3(3)	121.1
C26−C27−C28	120.1(3)	119.8
C29−C28−C27	120.4(3)	120.9
C28−C29−C30	120.7(3)	120.8
C29−C30−C21	124.3(2)	124.5
C29−C30−C25	118.5(2)	118.2
C21−C30−C25	117.2(2)	117.3
N2−N1−Ru1	116.16(16)	116.3
C21−N2−N1	111.6(2)	112.7
C21−N2−Ru2	112.84(16)	115.4
N1−N2−Ru2	115.98(15)	110.5
Ru2−Cl2−Ru1	110.47(3)	109.72
N1−Ru1−C2	91.60(10)	95.74
N1−Ru1−C6	153.87(12)	155.69
C2−Ru1−C6	67.92(12)	65.97
N1−Ru1−C5	158.36(10)	159.11
C2−Ru1−C5	80.26(12)	77.66
C6−Ru1−C5	37.40(12)	36.68
N1−Ru1−C3	93.63(10)	97.57
C2−Ru1−C3	38.35(11)	37.04
C6−Ru1−C3	80.40(11)	77.66
C5−Ru1−C3	67.58(11)	65.18
N1−Ru1−C1	115.85(11)	118.90
C2−Ru1−C1	37.52(12)	36.51
C6−Ru1−C1	38.33(13)	37.28
C5−Ru1−C1	68.56(13)	66.61
C3−Ru1−C1	68.73(12)	66.36
N1−Ru1−C4	120.19(10)	122.40
C2−Ru1−C4	68.55(11)	65.79
C6−Ru1−C4	68.61(11)	66.29
C5−Ru1−C4	38.18(11)	36.83
C3−Ru1−C4	37.25(10)	35.70
C1−Ru1−C4	81.87(11)	78.60
N1−Ru1−Cl1	83.98(6)	84.86
C2−Ru1−Cl1	118.71(9)	119.47
C6−Ru1−Cl1	91.70(8)	90.47
C5−Ru1−Cl1	117.53(8)	115.73
C3−Ru−Cl1	157.00(8)	156.45

C1−Ru1−Cl1	91.68(9)	91.87
C4−Ru1−Cl1	155.43(7)	152.48
N1−Ru1−Cl2	85.57(7)	82.88
C2−Ru1−Cl2	154.54(9)	152.28
C6−Ru1−Cl2	119.94(10)	120.86
C5−Ru−Cl2	93.21(9)	93.79
C3−Ru1−Cl2	116.50(8)	115.47
C1−Ru1−Cl2	158.17(10)	158.14
C4−Ru1−Cl2	91.09(8)	91.49
Cl1−Ru1−Cl2	86.19(2)	88.08
C12−Ru2−C16	67.60(12)	65.86
C12−Ru2−C13	37.58(12)	36.69
C16−Ru2−C13	79.90(12)	77.58
C12−Ru2−N2	150.19(11)	157.52
C16−Ru2−N2	92.39(10)	96.80
C13−Ru2−N2	163.77(10)	157.74
C16−Ru2−C15	37.95(11)	36.99
C13−Ru2−C15	67.55(11)	65.25
N2−Ru2−C15	97.62(9)	97.21
C12−Ru2−C11	38.20(13)	37.27
C16−Ru2−C11	37.40(12)	36.40
C13−Ru2−C11	68.57(13)	66.60
N2−Ru2−C11	113.25(11)	120.59
C15−Ru2−C11	68.49(12)	66.29
C12−Ru2−C14	68.25(12)	66.46
C16−Ru2−C14	67.89(11)	65.87
C13−Ru2−C14	37.84(10)	36.93
N2−Ru2−C14	125.96(9)	121.10
C15−Ru2−C14	37.24(10)	35.83
C11−Ru2−C14	81.33(12)	78.74
C12−Ru2−Cl3	88.23(9)	90.46
C16−Ru2−Cl3	125.23(9)	118.32
C13−Ru2−Cl3	110.26(8)	116.31
N2−Ru2−Cl3	85.87(6)	85.45
C15−Ru2−Cl3	162.65(8)	155.28
C11−Ru2−Cl3	94.47(9)	91.13
C14−Ru2−Cl3	147.01(7)	153.20
C12−Ru2−Cl2	124.60(10)	118.51
C16−Ru2−Cl2	148.68(9)	153.66
C13−Ru2−Cl2	95.10(9)	92.19
N2−Ru2−Cl2	84.06(6)	83.49

C15−Ru2−Cl2	111.58(8)	116.71
C11−Ru2−Cl2	162.67(10)	155.77
C14−Ru2−Cl2	89.14(8)	91.33
Cl3−Ru2−Cl2	85.65(2)	88.00
R		0.994
MAE [°]		1.66

Table S3. Experimental and calculated chemical shifts (the numbering scheme follows Figure 2)

H atom	Experimental [ppm]	Theoretical [ppm]	C atom	Experimental [ppm]	Theoretical [ppm]
CH_3 (isopropyl)	1.05	1.34	C10	18.35	18.13
CH_3 (isopropyl)	1.28	1.47	C17	18.46	19.08
CH_3 (isopropyl)	1.29	1.20	C19	21.15	19.24
CH_3 (isopropyl)	1.34	1.37	C7	22.07	19.59
CH_3 (methyl)	1.88	1.95	C9	22.71	26.85
CH_3 (methyl)	2.15	1.73	C20	22.75	27.30
CH (isopropyl)	2.66	2.86	C18	31.79	34.65
CH (isopropyl)	2.91	2.97	C8	32.02	35.47
C(15)H	4.26	4.42	C17	78.57	76.20
C(16)H	4.98	4.77	C3	81.20	78.00
C(3)H	5.26	4.77	C2	82.07	83.02
C(2)H	5.38	4.77	C16	83.16	83.66
C(12)H	5.52	5.06	C5	83.20	84.97
C(13)H	5.52	5.34	C13	84.59	87.90
C(5)H	5.68	5.19	C15	84.62	88.21
C(6)H	5.68	5.47	C6	85.95	89.96
C(23)H	7.72	7.79	C11	97.42	99.47
C(27)H	7.67	7.94	C1	98.42	101.30
C(22)H	7.8	8.09	C14	106.98	114.48
C(28)H	8.29	8.09	C22	107.52	114.61
C(24)h	7.99	8.26	C4	121.16	115.57
C(26)H	8.1	8.32	C29	121.40	116.66
C(29)H	8.56	8.57	C30	125.27	119.85
R		0.994	C23	127.15	121.22
MAE [ppm]		0.24	C27	128.35	123.51
			C28	128.89	123.98
			C24	129.80	126.65
			C26	130.69	126.65
			C25	135.21	131.01
			C21	147.70	143.60
			R		0.996
			MAE [ppm]		3.54

Table S4. Calculated chemical shifts at PBE0/6-31+G(d,p)(H,C,N,Cl)/LanL2DZ(Ru) (lev.1) and PBE0/6-311++G(d,p)(H, C, N, Cl)/LanL2DZ(Ru) (lev.2) (the numbering scheme follows Figure 2)

H atom	lev.1 [ppm]	lev.2 [ppm]	C atom	lev.l [ppm]	lev.2 [ppm]
CH_3 (isopropyl)	1.18	1.12	C10	19.03	18.28
CH_3 (isopropyl)	1.20	1.24	C17	20.80	19.51
CH_3 (isopropyl)	1.31	1.27	C19	19.07	18.67
CH_3 (isopropyl)	1.37	1.34	C7	21.57	20.06
CH_3 (methyl)	1.73	2.02	C9	27.81	26.92
CH_3 (methyl)	2.06	2.30	C20	27.97	27.03
CH (isopropyl)	2.99	3.10	C18	38.80	37.47
CH (isopropyl)	2.99	3.10	C8	38.89	37.24
C(16)H	4.88	4.99	C17	78.45	79.88
C(3)H	4.81	4.96	C3	80.56	80.64
C(2)H	4.74	8.86	C2	84.24	84.28
C(12)H	4.93	4.99	C16	83.54	84.46
C(13)H	4.93	4.99	C5	85.77	86.58
C(5)H	5.22	5.36	C13	89.70	89.59
C(6)H	5.13	5.20	C15	87.92	88.30
C(23)H	7.78	8.01	C6	89.77	90.11
C(27)H	7.93	8.13	C11	89.77	101.43
C(22)H	8.13	8.37	C1	103.25	101.93
C(28)H	8.01	8.17	C14	115.39	115.00
C(24)h	8.13	8.37	C22	118.22	118.16
C(26)H	8.21	8.44	C4	117.68	116.21
C(29)H	8.45	8.75	C29	120.27	119.52
R	0.994	0.993	C30	122.36	121.43
MAE [ppm]	0.26	0.23	C23	125.74	125.83
			C27	127.78	127.52
			C28	127.93	127.56
			C24	131.03	129.91
			C26	130.63	129.82
			C25	134.00	132.32
			C21	147.65	146.71
			R	0.996	0.996
			MAE [ppm]	3.08	3.05
